# Mutational Signatures Driven by Epigenetic Determinants Enable the Stratification of Patients with Gastric Cancer for Therapeutic Intervention

**DOI:** 10.3390/cancers13030490

**Published:** 2021-01-27

**Authors:** Jaqueline Ramalho Buttura, Monize Nakamoto Provisor Santos, Renan Valieris, Rodrigo Duarte Drummond, Alexandre Defelicibus, João Paulo Lima, Vinicius Fernando Calsavara, Helano Carioca Freitas, Vladmir C. Cordeiro de Lima, Thais Fernanda Bartelli, Marc Wiedner, Rafael Rosales, Kenneth John Gollob, Joanna Loizou, Emmanuel Dias-Neto, Diana Noronha Nunes, Israel Tojal da Silva

**Affiliations:** 1 Laboratory of Bioinformatics and Computational Biology, A.C. Camargo Cancer Center, São Paulo 01508-010, Brazil; jaqueline.ramalho@accamargo.org.br (J.R.B.); monize.santos@grupofleury.com.br (M.N.P.S.); renan.valieris@accamargo.org.br (R.V.); rdrummond@accamargo.org.br (R.D.D.); alexandre.defelicibus@accamargo.org.br (A.D.); joao.lima@accamargo.org.br (J.P.L.); 2 Department of Genomics, Fleury Group, São Paulo 04344-070, Brazil; 3 Department of Statistics and Epidemiology, A.C. Camargo Cancer Center, São Paulo 01508-010, Brazil; vinicius.calsavara@cshs.org; 4 Medical Oncology Department, A.C. Camargo Cancer Center, São Paulo 01508-010, Brazil; helano.freitas@accamargo.org.br (H.C.F.); vladmir.lima@oncologiador.com.br (V.C.C.d.L.); 5 Laboratory of Medical Genomics, A.C. Camargo Cancer Center, São Paulo 01508-010, Brazil; thais.bartelli@accamargo.org.br (T.F.B.); emmanuel@accamargo.org.br (E.D.-N.); dnoronha@accamargo.org.br (D.N.N.); 6 Translational Immuno-Oncology Group, A.C. Camargo Cancer Center, São Paulo 01508-010, Brazil; kenneth.gollob@accamargo.org.br; 7 CeMM Research Center for Molecular Medicine of the Austrian Academy of Sciences, 1090 Vienna, Austria; mwiedner@cemm.oeaw.ac.at (M.W.); jloizou@cemm.oeaw.ac.at (J.L.); 8 Department of Mathematics and Computer Science, University of São Paulo, Ribeirão Preto 14049-900, Brazil; rrosales@usp.br; 9 Department of Medicine, Institute of Cancer Research, Medical University of Vienna and Comprehensive Cancer Center, 1090 Vienna, Austria; 10 Laboratory of Neurosciences, Institute of Psychiatry, University of São Paulo, São Paulo 05403-903, Brazil

**Keywords:** mutational signature, gastric cancer, DNA mismatch repair, prognosis

## Abstract

**Simple Summary:**

Mutational signatures due to DNA mismatch repair deficiency (dMMR) is common in many cancers. However, the prognostic value of dMMR-associated mutational signatures remains to be assessed. Here, we performed a *de novo* extraction of mutational signatures in a cohort of 787 patients with gastric cancer. We detected three dMMR-related signatures, one of which clearly discriminates tumors with *MLH1* gene silencing through hypermethylation of its promoter. We showed evidence that classification based on mutational signature exposure can be used to identify groups of patients with common clinical, immunological, and mutational features related directly to better prognosis.

**Abstract:**

DNA mismatch repair deficiency (dMMR) is associated with the microsatellite instability (MSI) phenotype and leads to increased mutation load, which in turn may impact anti-tumor immune responses and treatment effectiveness. Various mutational signatures directly linked to dMMR have been described for primary cancers. To investigate which mutational signatures are associated with prognosis in gastric cancer, we performed a *de novo* extraction of mutational signatures in a cohort of 787 patients. We detected three dMMR-related signatures, one of which clearly discriminates tumors with *MLH1* gene silencing caused by promoter hypermethylation (area under the curve = 98%). We then demonstrated that samples with the highest exposure of this signature share features related to better prognosis, encompassing clinical and molecular aspects and altered immune infiltrate composition. Overall, the assessment of the prognostic value and of the impact of modifications in MMR-related genes on shaping specific dMMR mutational signatures provides evidence that classification based on mutational signature exposure enables prognosis stratification.

## 1. Introduction

Cancer results from the sequential accumulation of DNA alterations, including single-nucleotide mutations [1] that arise from various endogenous and exogenous processes [2]. Distinct DNA-damaging processes leave characteristic nucleotide base-change footprints known as mutational signatures [3]. Researchers have extracted distinct mutational signatures by examining large sets of human cancer genomes, and some of these signatures have been registered in the COSMIC (CS) database (http://cancer.sanger.ac.uk/cosmic/signatures) [4]. This pan-cancer analysis revealed the significant heterogeneity of operational mutational processes, which encompass mutation-triggering events as diverse as the off-target activity of the *AID*/*APOBEC* family of cytidine deaminases, exposure to ultraviolet light, tobacco smoking, and defective DNA mismatch repair (dMMR) [5,6].

Collectively, the understanding of the mechanistic basis and etiology of mutational signatures may provide clues for cancer diagnosis and have prognostic value [7]. For example, six mutational signatures have been associated with the *BRAC1/2* gene dysfunction, and most likely are predictive of the response to treatment with poly-ADP ribose polymerase (PARP) inhibitors [8]. Homologous recombination repair (HRR) deficiency features based on these signatures allowed the prediction of BRCAness in patients with breast cancer with a 98.7% sensitivity [8]. Additionally, given that nucleotide excision repair (NER)-deficient tumors are more sensitive to certain treatments, somatic variations in the *ERCC2* gene, which encodes a key protein of the NER pathway, have also been linked to characteristic mutational signatures [9,10]. Other mutational processes have been associated with the harboring of biallelic *MUTYH* germline mutations [11], which may indicate deficient base excision repair (BER). Affected patients are eligible for genetic counseling [12] and might benefit from immunotherapy [13].

In addition to the HRR, NER, and BER repair pathways, another mechanism underlying oncogenic genomic variations occurs in tumors with impaired DNA mismatch repair (MMR) which harbor elevated frequencies of single-nucleotide variants (SNVs) and exceptionally high indel rates [14]. Recent studies have demonstrated that various tumors with mismatch repair deficiency (dMMR; glioblastomas and gastrointestinal, endometrial, and prostate tumors) are more responsive to programmed cell death protein 1 (PD1) immune checkpoint inhibitors than MMR-proficient tumors are [15,16,17]. The microsatellite instability (MSI) phenotype arises mainly because of the dMMR mechanism [18], and thus serves together with the immunohistochemical detection of MMR genes (e.g., *MLH1* and *MSH2*) as a biomarker for the identification of patients with MSI/dMMR and a guide for decisions about their clinical treatment.

A set of four mutational signatures (CS-6, CS-15, CS-20, and CS-26) has been associated with the dMMR and/or MSI phenotypes, mainly in the context of colorectal cancer and Lynch syndrome. CS-6 and CS-15 have also been described in the context of gastric cancer [19]. Furthermore, an improved prognosis of gastric cancer has been associated with dMMR/MSI status, without consideration of the mutational signature landscape [18,19]. It is thought that mutational signatures arise from multiple changes in pathway component events [3,4], and thus its evaluation as a classifier may be more informative than a unique clinical or molecular feature, which allows grouping patients with similar phenotypes based on their mutational profiles.

In this study, we sought to identify mutational signatures associated with the prognosis of gastric cancer and determine the significance of molecular events in MMR genes that shape these signatures in MMR-deficient gastric adenocarcinomas. The prognostic value of the presence of these signatures was evaluated in a cohort of 787 patients with gastric cancer, including 439 patients whose data is registered in The Cancer Genome Atlas (TCGA) and a validation cohort of 170 patients with gastric cancer [20]. We further investigated whether local tumor immune responses and prognoses varied according to the dMMR exposure load. The stratification of patients with gastric cancer by dMMR mutational signature appears to be related to tumor microenvironment and molecular features predictive of the responsiveness to immune checkpoint blockade. Further studies are recommended to confirm this in clinical practice.

## 2. Results

### 2.1. Mutational Signatures

**Figure 1:**
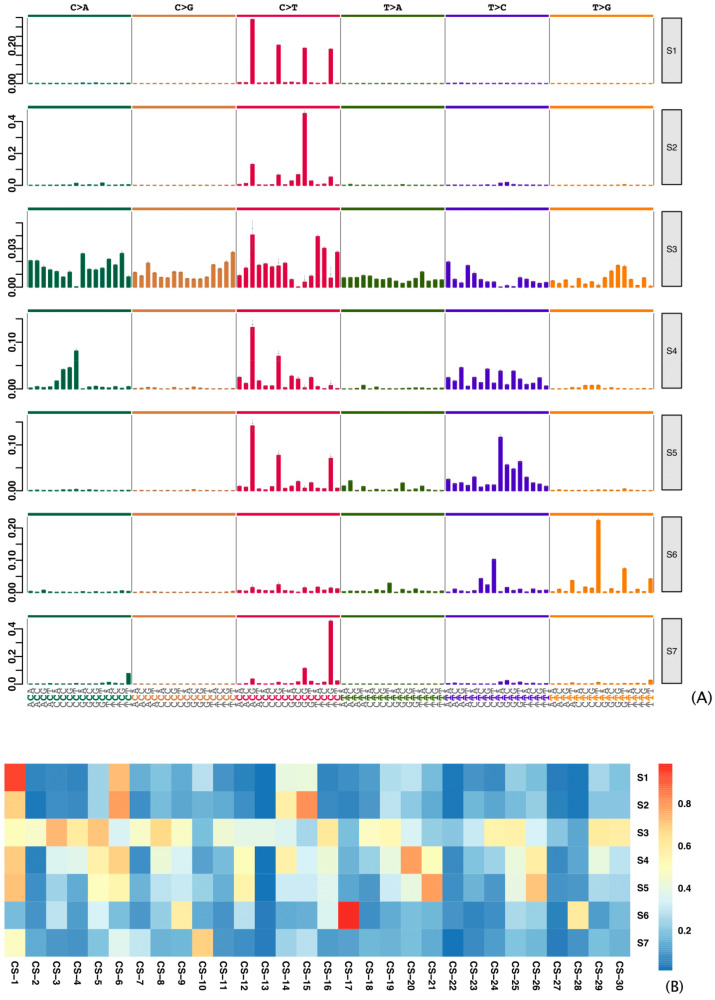
De novo mutational signatures (Ss) in gastric cancer samples. (**A**) Mutational signatures in gastric cancer samples from 787 patients, extracted from The Cancer Genome Atlas, International Cancer Genome Consortium, and cbioPortal cohorts. (**B**) Heatmap of cosine similarities between de novo mutational signatures and COSMIC signatures (CSs).

Using signer [21] analysis to estimate de novo mutational signatures across three gastric cancer cohorts, we identified seven mutational signatures (S1–7; Figure 1A) which are related to signatures described in the CS database (CSs), as reflected by cosine similarity scores (Figure 1B). S1 (related to CS-1) is associated with endogenous mutational processes initiated by the spontaneous deamination of 5-methylcytosine; S2, S4, and S5 (related to CS-6 and CS-15, CS-20, and CS-21 and CS-26, respectively) are associated with dMMR and/or MSI; S3 (related to CS-3) is associated with the failure of DNA double-strand break repair by homologous recombination; S6 (related to CS-17) is of unknown etiology; and S7 (related to CS-10) is associated with error-prone polymerase activity in the catalytic subunit of DNA polymerase epsilon (POLE).

CS-3 (S3; Figure 1 and Figure S1) was the predominant signature, and the observations in this study support previous characterizations of it in gastric cancer samples as having a very high prevalence of small indels and base substitutions due to the failure of DNA double-strand break repair by homologous recombination [19]. This finding suggests that 7–12% of patients with gastric cancers could benefit from platinum or PARP inhibitor therapy. Notably, however, some patients not exposed to CS-3 were found to be highly exposed to signatures associated with dMMR (S2, S4, and S5; Figure S1). This distinct group of patients harbors features that might have prognostic relevance; we investigated this possibility further.

### 2.2. dMMR Signatures and Prognostic Features

To assess whether dMMR signature exposure had prognostic value in patients with gastric cancer, we first evaluated the influences of each mutational signature exposure and its main possible clinical and molecular prognostic features—such as age at diagnosis, ethnicity, tumor pathological stage, Lauren classification, anatomic site, tumor mutational burden (TMB), and MSI status—on overall survival (OS) by fitting a simple Cox regression model (Figure S2). We then fitted a multiple Cox regression model to the dataset using prognostic features with significant *p* values (*p* < 0.05) in the simple model (Figure S2). Data from 584 patients with gastric cancer and no metastasis at diagnosis for whom vital status information (alive/dead) was available were included in simple and multiple Cox regression models. The median and mean follow-up durations for these patients were 28.9 months (95% confidence interval (CI), 25.8–32.1 months) and 36.2 months (95% CI, 32.9–39.5 months), respectively.

**Figure 2:**
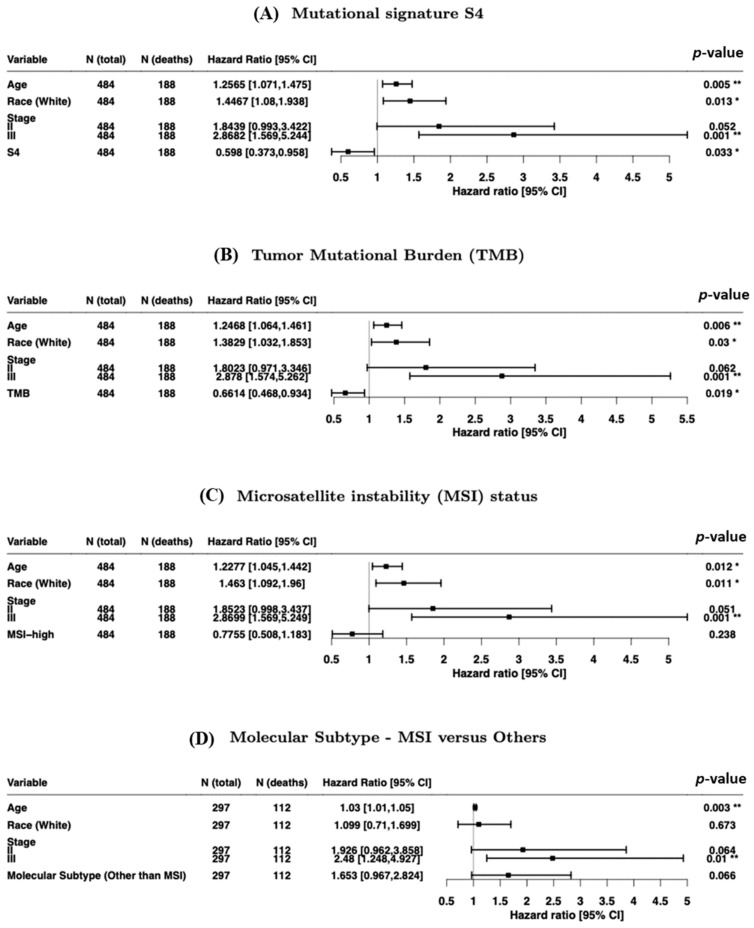
Forest plots of hazard ratios for overall survival (multiple Cox regression models) for (**A**) mutational signature S4, (**B**) the tumor mutational burden (TMB), and (**C**) microsatellite instability (MSI) status and (**D**) MSI molecular subtype. CI, confidence interval. * *p* < 0.05, ** *p* < 0.01.

Relative to other dMMR signatures, S4 exposure burden was associated with an improved OS (hazard ratio (HR) = 0.59; 95% CI, 0.37–0.96; Figure 2A and Figure S3). Thus, we focused on S4, which has the potential to offer important, clinically actionable information for prognosis.

Our analysis also revealed that higher TMBs were associated with improved OS (HR = 0.66; 95% CI, 0.46–0.93; Figure 2B), consistent with previous findings [22]. OS was not associated with high microsatellite instability (MSI-H) status or MSI molecular subtype (Figure 2C,D). The calibration curves for 2-year OS indicated that all the models were adequate (Figure S4).

We used the “maxstat” function in R to define groups according to S4 exposure. The optimal cut-point was in the highest quartile (Q3); thus, patients with S4 exposure ≥ the Q3 level were allocated to the S4^high^ status, and all other patients (<Q3 level) were allocated to the S4^low^ group. Survival curves differed significantly between these groups (*p* = 0.03, Log-rank test); median OS durations were 72 months (95% CI, 48.0 months–∞) in the S4^high^ group and 37 months (95% CI, 28.0–68.0 months) in the S4^low^ group considering the whole follow-up (Figure S5, throughout 5-years overall survival). Next, we used an independent gastric cancer cohort to validate that S4^high^ conferred a survival benefit. In the Kaplan–Meier analysis, the median OS duration had not been reached at 5 years (95% CI, 38.2 months–∞) in the S4^high^ group and was 48 months (95% CI, 21.3 months–∞) in the S4^low^ group, supporting the presence of a trend toward a survival benefit for S4^high^ (Figure S6).

### 2.3. Clinical and Molecular Features in the S4 dMMR Groups

We next further examined the clinical and molecular features of the S4^high^ and S4^low^ groups. We first determined whether S4 exposure was represented equally in the three cohorts. To avoid performing statistical tests with different numbers of samples, a subsampling procedure was applied; 24 samples were selected randomly from each cohort and the Kruskal–Wallis test was applied to this sub cohort. The procedure was repeated 1000 times, and all the *p* values were >0.05, indicating that the S4 exposure was similar in all cohorts. These results reinforce the prognostic value of S4 signature independent of ethnic background.

**Figure 3:**
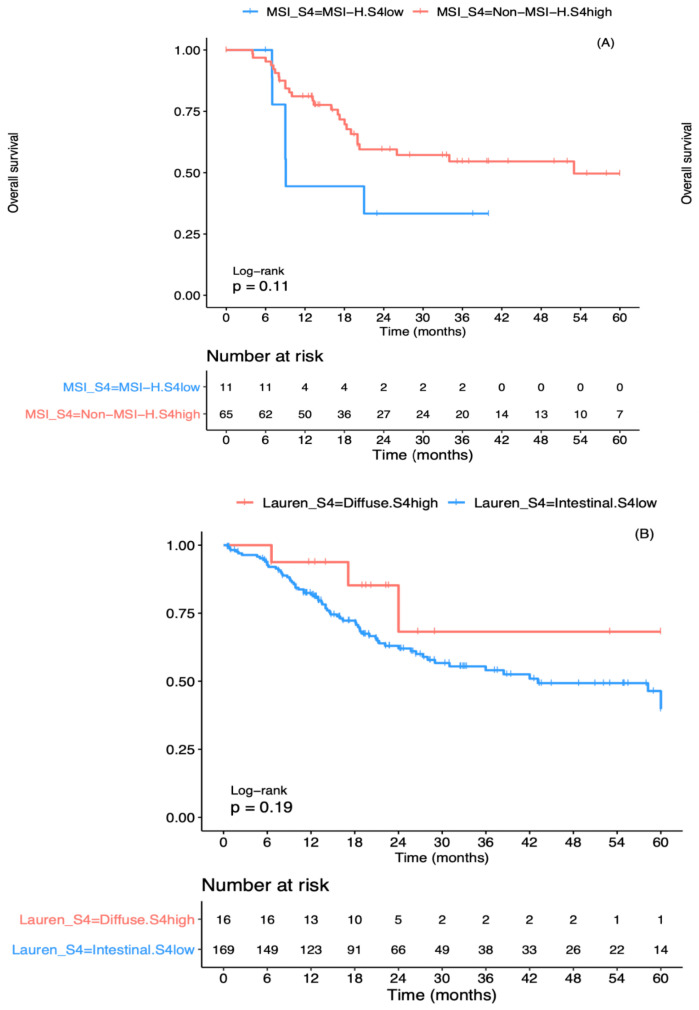
Five-year overall survival for subgroups of the mutational signature S4^high^ and S4^low^ groups in the public cohort. (**A**) Comparison of cases with high microsatellite instability (MSI-H) and in the S4^low^ group with non-MSI-H cases in the S4^high^ group. (**B**) Comparison of cases with diffuse histology in the S4^high^ group with those with intestinal histology in the S4^low^ group. Log rank test was used to compare the survival distributions of two groups.

The clinical features associated previously with the improved prognosis of gastric cancer, such as distal anatomic site and intestinal histology [23], were also enriched in the S4^high^ group (Table 1). This group also showed a significantly greater occurrence of clinical variables known to be associated with worse gastric cancer prognosis, such as cardia/proximal anatomic site, diffuse histology, positive lymph-node metastasis (stage N+), and advanced pathological stage (III and IV) [23] (Table 1). In addition, the predicted MSI-H status, MSI, and POLE molecular subtypes were enriched in the S4^high^ group, whereas the genomically stable (GS) and chromosomal instability (CIN) molecular subtypes were enriched in the S4^low^ group (Table 1). Most (*n* = 119/160 (74%)) of the MSI-H cases were in the S4^high^ group, but the S4^low^ group unexpectedly contained a small proportion of such cases and the S4^high^ group contained non-MSI-H cases (Table 1). Survival curves revealed a trend for worse prognosis for MSI-H cases in the S4^low^ group (median OS duration, 9.07 months; 95% CI, 9.0 months–∞) than non-MSI-H cases in the S4^high^ group (median OS, 53 months; 95% CI, 20.0 months–∞; Figure 3A). The prognosis seems to be better for cases with the diffuse histological subtype in the S4^high^ group (median OS not reached; 95% CI, 24.0 months–∞) than the intestinal histological subtype in the S4^low^ group (median OS 43.1 months; 95% CI, 28.0 months–∞; Figure 3B). These results indicate that classification according to mutational signatures improved the stratification of patients within the prognostic groups, independent of their previous clinical or molecular classification.

**Figure 4:**
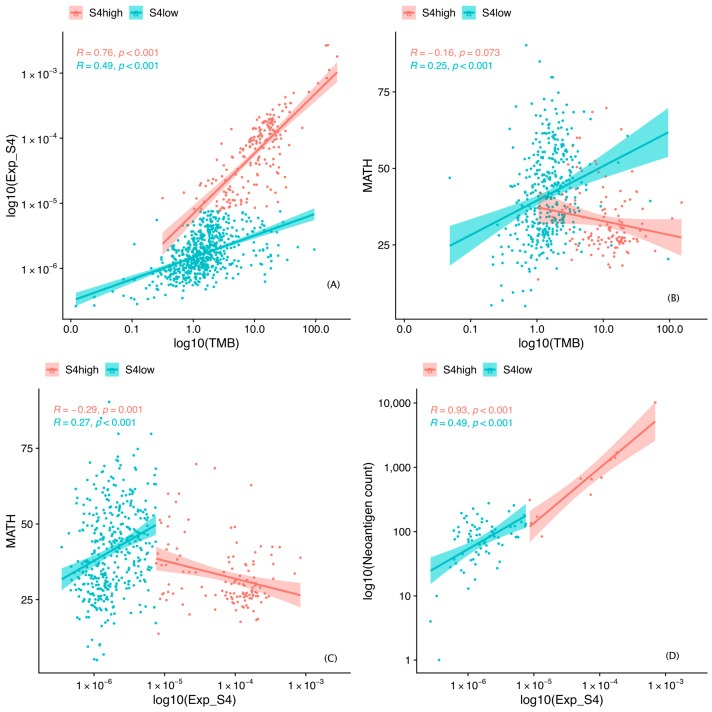
Scatter plots of Spearman correlation between molecular features. S4, mutational signature 4. TMB, tumor mutational burden; MATH, mutant-allele tumor heterogeneity.

We also examined the tumor heterogeneity, TMB, and neoantigen count. Tumor heterogeneity was evaluated quantitatively by the examination of the distribution of allele frequencies and the calculation of mutant-allele tumor heterogeneity (MATH) scores [24]. We then performed a correlation analysis that incorporated the MATH score, S4 exposure, and TMB (Figure 4). Correlations of the MATH score with TMB and S4 exposure were negative in the S4^high^ group and positive in the S4^low^ group. In addition, the MATH scores were higher in the S4^low^ group than in the S4^high^ group (*p* = 3.711 × 10^−12^, Mann–Whitney *U* test). The TMB and neoantigen load correlated positively with S4 exposure in both groups (Figure 4).

These findings suggest that tumors with high S4 exposure were more homogeneous in the S4^high^ group, and that reduced tumor heterogeneity together with a high TMB and high neoantigen load is determinant of a good prognosis.

### 2.4. S4 is Associated with Epigenetic Changes and Mutational Background in MMR Genes

To investigate the mechanisms underlying S4, we evaluated the epigenetic context and mutational landscape of MMR genes in the S4^high^ and S4^low^ groups. Although the genes associated with dMMR have been identified, the underlying main genetic modifications that lead to each dMMR signature remain poorly characterized.

**Figure 5:**
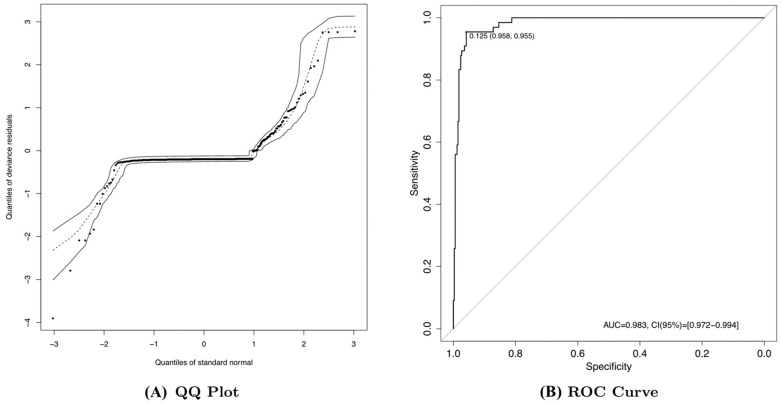
Performance and power discrimination of the logistic model. (**A**) Q–Q plot showing that the propensity score model was adequately calibrated, according to the Hosmer–Lemeshow test. (**B**) Summary of the receiver operating characteristic (ROC) curve showing the power of discrimination for *MLH1* methylation in mutational signature S4. AUC, area under the receiver operating characteristic curve; CI, confidence interval.

To improve our understanding of the determinant changes that influence the dMMR signatures detected in this study, we first searched for epigenetic changes in the MMR genes. In line with previous findings [25,26], we observed the down-regulation of *MLH1* expression, driven by the hypermethylation of its promoter (Figure S7, rho = −0.81, *p*-value < 0.001). To further assess the manner in which mutational exposure is associated with epigenetic changes in the *MLH1* gene, simple and multiple logistic regression models were fitted to the dataset (Table 2). S4 exposure burden was associated with an increased chance of *MLH1* promoter methylation (h*MLH1*; odds ratio (OR) = 22.561; 95% CI, 7.909–64.353) and S5 exposure burden was associated with a decreased chance of such methylation (OR = 0.107; 95% CI, 0.048–0.238); no such association was found for the S2 exposure burden. This model showed adequate to good performance (Hosmer–Lemeshow goodness-of-fit test: χ^2^(8) = 10.257, *p* = 0.247; Brier score = 0.0364; Figure 5A) and excellent discrimination power (area under the receiver operating characteristic curve (AUC) = 0.982; 95% CI, 0.971–0.994; Figure 5B). Using the Youden index, we determined that the best cutoff value was 0.125, which yielded a 95.45% sensitivity and a 95.82% specificity (Figure 5B).

We next looked for somatic and germline SNVs and indels in MMR genes (*LIG1*, *POLE*, *EXO1*, *MLH1*, *MLH3*, *MSH2*, *MSH3*, *MSH5*, *MSH6*, *PCNA*, *PMS1*, *PMS2*, *PMS2L3*, *PMS2L4*, *POLD1*, *POLD2*, *POLD3*, *POLD4*, and *SSBP1*). Six percent (*n* = 12/197) of patients harbored somatic variations in the *MLH1* gene; these patients were allocated to the S2^high^ and S4^high^ groups, and no such mutation was observed among patients in the respective S^low^ groups. Nine percent (*n* = 17/197) of patients in the S2^high^ group harbored somatic variations in the *MLH3* gene. We also found that only 8% of patients in the S5^high^ group (*n* = 8/100 considering TCGA cohort) harbored *MSH5* germline mutations. Overall, we observed few cases of mutated MMR genes in the S^high^ groups.

These observations suggest that germline SNVs, somatic SNVs, and indels are not the major modifications affecting MMR gene expression levels. hMLH1 was observed in almost 60% of individuals in the S4 group (*β* ≥ 0.3), considering the TCGA dataset. To validate this mechanism as a causative factor for S4, we examined genomic sequencing data from three HAP1 cell samples (two MLH1 knockout and one MLH1 wild type). We observed greater S4 exposure in the MLH1 knockout cell lines and greater S5 exposure in the parental cell line (Figure S8), which identifies the absence of MLH1 as a strong cause of the S4 mutational signature.

### 2.5. Somatic Changes Associated with S4

We further evaluated significantly mutated genes other than those related to MMR in the S4^high^ and S4^low^ groups. We observed increased numbers of SNVs and indels in the S4^high^ group; most mutations in the S4^low^ group were SNVs. These findings were expected, considering that MSI/dMMR harbors a mutator phenotype [14].

The significantly mutated [27] gene set in the S4^high^ group consisted of 102 genes. The most commonly mutated genes in this group were *ARID1A* (42%), *KMT2D* (35%), and *TP53* (31%), in accordance with previous findings [28]; 56 other genes presented mutations in at least 10% of patients (Table S1a). Somatic mutations in chromatin-regulating genes, such as *KMT2D* (also known as *MLL2*) and *ARID1A*, may be associated with improved survival [26]. Of the 24 significantly mutated genes identified in the S4^low^ group, 12 were oncogenes associated with tumor progression or tumor suppressor genes (*PIK3CA*, *KRAS*, *RHOA*, *CDH1*, *CTNNB1*, *ITGAV*, *SMAD4*, *TP53*, *CDKN2A*, *APC*, *PTEN*, and *PIK3R1*; Table S1c). These findings support previous reports of the occurrence of *APC*, *CTNNB1*, *SMAD4*, and *SMAD2* mutations among 215 non-hypermutated tumors from the TCGA cohort, with somatic mutations in *CDH1* and *RHOA* enriched in the GS and/or diffuse histologic subtypes [28], as seen in our S4^low^ group.

To assess other mechanisms underlying the remaining ~40% of mutations in the S4^high^ samples, we divided this group into hypermethylated and hypomethylated subgroups according to the *MLH1*-based methylation levels. The *TP53* gene was associated with hypomethylation in the S4^high^ group (OR = 0.314; 95% CI, 0.131–0.736; *p* = 0.006). However, in a comparison of all significantly mutated genes (MutSigCV analysis, *p* < 0.05) between the S4^high^ and S4^low^ groups, *TP53* was the only mutated gene associated with the S4^low^ group (OR = 0.428; 95% CI, 0.293–0.622; *p* = 3.542 × 10^−6^). An additional analysis of the somatic interactions (“somaticInteractions” function in the “maftools” package, available in R) revealed exclusive interactions of *TP53* in both groups, indicating different pathways underlying tumorigenesis for each group [29]. Additional studies are necessary to indicate a biomarker that encompasses both hyper and hypomethylated S4^high^ patients.

### 2.6. Tumor Microenvironment in S4 Groups

**Figure 6:**
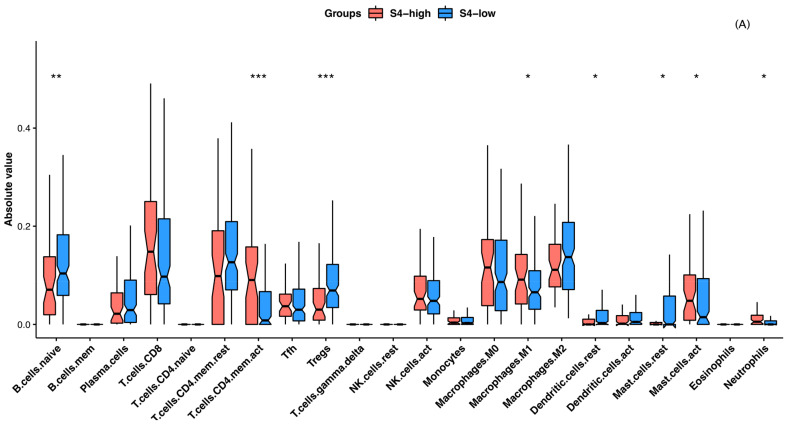

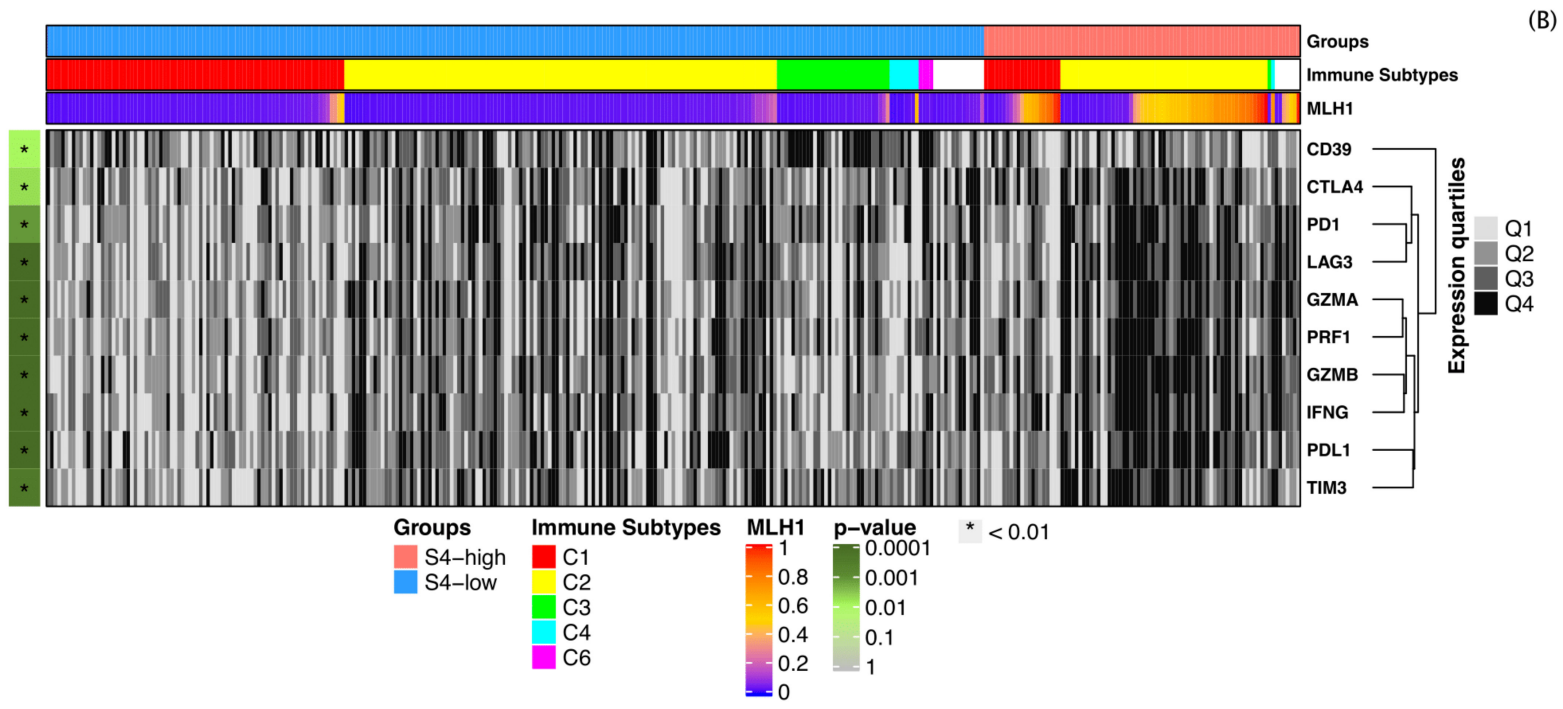
The main immunological features associated with the mutational signature (S)4^high^ and S4^low^ groups. (**A**) Boxplots showing the absolute quantification of immune infiltrate cells estimated by CIBERSORT. * *p* < 0.05, ** *p* < 0.01, *** *p* < 0.001, Mann–Whitney *U* test. (**B**) Heatmap of immune effector gene (cytotoxic and immune checkpoints) expression. Normalized gene expression levels for each marker gene were classified in quartiles. Q1, 0–25%; Q2, 25–50% (median); Q3, 50–75%; Q4, 75–100%. P values to the left of the heatmap for S4^high^ vs. S4^low^ expression were obtained using the Mann–Whitney *U* test. These comparisons are also shown as boxplots in Figure S9.

To investigate the association of the tumor microenvironment with the improved clinical outcomes seen in the S4^high^ group relative to the S4^low^ group, we determined the immune cell infiltrate and stromal cell compositions in the groups using biomarker gene expression methodologies. We found significantly greater proportions of infiltrating cytotoxic and pro-inflammatory immune cells, such as CD8+ central and effector memory T cells, CD4+ memory T cells, T helper 1 cells, gamma/delta T cells, natural killer (NK) cells, M1 macrophages, and plasmacytoid dendritic cells, in the S4^high^ group than in the S4^low^ group (Figure 6A and Figure S9). In contrast, immature and immune regulatory dendritic cells were more common in the S4^low^ group (Figure 6A and Figure S9).

To further characterize the immune regulatory environment associated with clinical outcomes, we compared the expression levels of key genes coding for immunoregulatory and effector molecules that have proven to be important for the control of many cancers [30,31]. Genes encoding the CD8+ T-cell–related cytolytic molecules granzyme A/B and perforin-1 (*GZMA* and *GZMB* and *PRF1*, respectively), the inflammatory T-cell response-related cytokine interferon (IFN)-γ (*IFNG*), other proinflammatory cytokines (*IL1B*, *IL6*, and *IL8*), and the NK cell killer-cell immunoglobulin-like receptor family, as well as T-cell activation marker genes (*IL2RA* and *ICOS*), showed greater expression in the S4^high^ than in the S4^low^ group (Figure 6B and Figure S10). No difference in the immunosuppression-related genes *TGFB1*, *IL-10*, and *FOXP3* was observed between groups, whereas the expression of *ENTPD1* (encoding CD39, a protein associated with T regulatory cell (Treg) immunosuppression activity) [32] was greater in the S4^low^ than in the S4^high^ group (Figure 6B and Figure S10). Importantly, the expression of immune checkpoint inhibitor genes (*PDCD1* for the PD1 receptor; *CD274* for programmed death ligand (PDL)-1; *PDCD1LG2* for PD-L2; *HAVCR2* for TIM3, *LAG3*, and *CTLA4*) was also greater in the S4^high^ group (Figure 6B and Figure S10), suggesting a relationship with a more immunologically activated tumor microenvironment [33]. The expression of *HLA*, antigen-processing, and presentation-related genes (e.g., *CD86, B2M, HLA* class II genes, *HLA-E, HLA-C*, *TAP1*, and *TAP2*) was also greater in the S4^high^ group (Figure S10). The observation of previously characterized immune subtypes [34] reinforces the finding that the tumor microenvironment in the S4^high^ group is more immunologically active, composed predominantly of the C2 (IFN-γ) immune subtype, with a significantly greater proportion of this subtype than that observed in the S4^low^ (Table 1, Figure 6B). This subtype has been associated with highly mutated tumors [34]. On the other hand, the S4^low^ group displayed greater proportions of the C3 (inflammatory) and C1 (wound healing) immune subtypes [34] (Table 1, Figure 6B). The C2 immune subtype appeared to be less activated in the S4^low^ group than in the S4^high^ group, with a reduced relative gene expression of immune effector molecules (Figure 6B). Together, these findings indicate that the immune microenvironment in the S4^high^ group is strongly activated relative to that in the S4^low^ group.

In the evaluation of stromal cells by xCell analysis, scores for myocytes, chondrocytes, hematopoietic stem cells, endothelial cells (microvascular and lymphatic), and fibroblasts (pericytes and mesangial cells) were higher in S4^low^ group (Figure S9). Pericytes and endothelial cells are important cellular components of the tumor microenvironment that have been associated with the worst prognosis, considering the high risks of angiogenic events and metastasis [35,36,37]. Overall, the stromal score in the S4^low^ group was higher than that in the S4^high^ group, demonstrating an association with the worst prognosis (Figure S9).

## 3. Discussion

In this study, we conducted a comprehensive and integrated analysis of the impacts of MMR-related gene alterations on specific mutational signatures associated with gastric cancer. We present evidence that the identification of dMMR can be used not only for MSI phenotype classification, but also as a potential indicator of prognosis besides assembling potential patients with a peculiar tumor microenvironment that would respond to immune checkpoint inhibitors.

We performed a de novo extraction of mutational signatures based on somatic SNVs across four whole-exome sequencing cohorts, encompassing 787 gastric cancer samples derived mainly from populations of European and Asian descent. We found seven mutational signatures, three of which were related to dMMR. S4, related to the previously described CS-20, was the only dMMR signature with significant prognostic value; this value was validated in a local cohort of patients with gastric cancer, which was distinct in terms of molecular ancestry and some clinical and molecular features (e.g., Lauren’s histology and tumor heterogeneity). Diffuse/mixed histology was predominant in this cohort, whereas the public cohorts were enriched in the intestinal subtype. Furthermore, the S4^low^ group in this independent cohort was less heterogeneous than the S4^low^ group from the public cohorts and the S4^high^ groups from both cohorts. Nevertheless, patients in the S4^high^ groups, including that of this cohort, had a better prognosis.

The main mechanism associated with MMR impairment in samples with S4 exposure seemed to be h*MLH1*. The disruption of *MLH1* in vitro using a CRISPR/Cas9 assay reproduced CS-20 [38], which resembles S4. In this study, we identified an endogenous epigenetic mechanism for this signature in patients with gastric cancer. We also reproduced S4 in an isogenic cell model in which *MLH1* knockout cells had a high S4 exposure. Thus, we conclude that the loss of *MLH1* gene expression due to promoter hypermethylation and mutagenesis loss of function result in the same mutational signature.

The CpG island methylator phenotype is a well-documented early event in tumorigenesis, preceding h*MLH1* in solid tumors, which in turn drives the MSI-H phenotype [25,26,28]. In contrast, the low MSI and microsatellite stability gastric carcinoma subtypes have unmethylated *MLH1* promoters and regular MLH1 activity [25]. In this study, we observed that most MSI-H cases were in the S4^high^ group, but that a small fraction of these cases were in the S4^low^ group. This observation is in line with previous reporting that about one fourth of MSI-H cases have distinct molecular features and poor prognoses [39]. In addition, the presence of non–MSI-H cases in our S4^high^ group shows that mutational signature exposure can be used to cluster samples independently of their MSI status. Furthermore, we identified a few cases (4%) in the S4^high^ group that did not present h*MLH1,* but carried somatic mutations in the *MLH1* gene that apparently lead to a loss of function of the encoded protein. Thus, we demonstrated that h*MLH1* is the main mechanism driving S4 (CS-20) in gastric cancer. Furthermore, we observed that patients without h*MLH1* harbor *TP53* mutations, suggesting another mechanism associated with dMMR signatures in this subset of patients.

We also demonstrated a strong correlation between S4 exposure and the TMB, which showed significant prognostic value for survival. Hypermutated tumors have been shown to have a better prognoses and good responses to immunotherapy, apparently due to neoantigen enrichment and intrinsic antitumor immune responses [40,41]. However, most thresholds for the identification of samples with high TMBs vary with tumor type, and some do not necessarily predict a better treatment response due to intratumoral heterogeneity [41,42,43]. Here, we observed that most mutations associated with the S4^high^ signature are clonal, which is important for the prediction of better treatment.

High degrees of intratumoral heterogeneity have been associated with incomplete responses to therapy, higher relapse rates, and poor clinical outcomes [44,45]. The increased genomic instability observed in MSI/dMMR and CIN tumors is the major driver of such heterogeneity [44,46]. However, the most unstable tumors (those with the greatest somatic SNV or copy number alteration burdens) are not the most intrinsically heterogeneous [46]. The greatest degree of intratumoral heterogeneity was found in tumors with relatively large numbers of somatic mutations and copy number alterations, which can be associated with exogenous mutagens, including those induced by viral infections and tobacco smoking. These tumors have large numbers of subclonal mutations related to late events and exhibit frequent chromosomal instability associated with the CIN subtype, *TP53* mutations, and APOBEC-related mutational signatures (previously related to the Epstein–Barr virus (EBV) gastric cancer subtype) [6,46]. Similarly, we observed greater tumor homogeneity in the S4^high^ than in the S4^low^ group. Thus, antitumor immune responses may be more effective in the S4^high^ group due to the intermediate to high TMBs and MSI and CIN molecular phenotypes, associated with lesser tumor heterogeneity. A recent meta-analysis revealed the importance of the MSI status to the treatment response in patients with gastric cancer, as its findings suggested that patients with MSI-H status may not benefit from perioperative or adjuvant therapy and could undergo surgery without these treatments [47].

Several studies have shown that the tumor microenvironment at diagnosis can be used to predict treatment response and clinical outcome [48,49]. The balance of inflammatory/cytotoxic immune cells with elements of an effective antitumor response, including regulatory cells and suppressor signals, may indicate which patients will intrinsically have such responses and thus a better prognosis.

The EBV and MSI subtypes of gastric cancer have been associated with greater immune infiltration and responsiveness to immunotherapy, as well as better prognoses [49]. In this study, we observed many elements indicating that the tumor microenvironment in the S4^high^ group was more active than that in the S4^low^ group, aggregating both cases of MSI and EBV molecular subtypes. In general, the absolute quantification of immune cell subtypes and differential gene expression using CIBERSORT [50] and the calculation of gene set enrichment analysis (GSEA) scores in xCell [51] revealed a greater activity of proinflammatory and cytotoxic cells, as well as more antigen processing and presentation, in the S4^high^ group. In contrast, although the S4^low^ group contained some immunogenic tumors, the predominant environment in this group was enriched in Treg lymphocytes and M2 macrophages, related to worse prognosis [48,49]. In addition, the S4^low^ group harbored stroma-enriched tumor microenvironments associated with poor prognosis [36].

## 4. Materials and Methods

### 4.1. Clinical and Genomic Data from Public Cohorts

Clinical and molecular data for 787 gastric adenocarcinoma samples were extracted from the following non-redundant public cohorts: (i) TCGA (STAD-US cohort, *n* = 439) (ii) cBioPortal (*n* = 226; composed by UHK (*n* = 19) [52], UHK/UHK_Pfizer (*n* = 100) [52], TMUCIH (*n* = 17) [53] and GACA-JP (*n* = 30) [54] cohorts), and (iii) the International Cancer Genome Consortium (ICGC, GACA-CN cohort, *n* = 122; Table S2 (available at https://dcc.icgc.org/projects/GACA-CN)). TCGA data were accessed on 4 October 2018 and correspond to the MC3 variant calling project, which is a comprehensive effort to detect consensus mutations and forms the basis of the Pan-Cancer Atlas initiative [55]. Data from the cBioPortal and ICGC cohorts, composed of patients of Asian descent, were last assessed on 9 January 2019.

Raw reads from matched non-tumor exomes including MMR genes were downloaded from the TCGA dataset and used to detect germline SNVs following the Genome Analysis Tool Kit’s (GATK’s) best practices for germline alteration calling. We also used filters to select mutations with a variant allele frequency ≥0.3 and a minimum depth coverage of 10 reads. The “dbNSFP_MetaLR_rankscore” annotation was used to filter out synonymous mutations (dbNSFP_MetaLR_rankscore ≤0.6). Fragment per kilobase million values from 380 TCGA stomach adenocarcinoma (STAD) samples were used to normalize the gene expression profiles. Methylation, quantified using *β* values (range, 0–1), was available for the TCGA cohort. We then used CpG sites to detect hypermethylated and hypomethylated MMR gene promoters (threshold, *β* ≥ 0.3). The baseline clinical features of the cases from each cohort are summarized in Table S3.

### 4.2. Clinical and Genomic Data from the Validation Cohort

Patients in the validation cohort were enrolled prospectively in an institutional study of the epidemiology and genomics of gastric adenocarcinomas in Brazil [20]. This study was approved by the local ethics committee, and all the participants provided written informed consent [20]. The clinical and molecular characteristics of the 170 patients in the validation cohort are provided in Table S4.

Genomic DNA was extracted from frozen tissues (*n* = 165) using the AllPrep DNA/RNA Mini Kit (Qiagen), QIASymphony THC 400 device (Qiagen), or phenol/chloroform/isoamyl alcohol precipitation. Genomic DNA was extracted from formalin-fixed paraffin-embedded tissues (*n* = 4) using the RecoverAll Total Nucleic Acid Isolation Kit (Thermo Fisher). One sample was from a gastric wash. Exome libraries were prepared using the Agilent SureSelect V6 kit and sequenced using Illumina platforms (HiSeq4000, 100 bp, *n* = 33; Novaseq, 150 bp, *n* = 137; paired-end reads for both). Somatic SNVs were called using an in-house pipeline following the GATK’s best practice guidelines [56], as described previously [6]. Briefly, the raw reads were aligned using the Burrows–Wheeler aligner with default settings to assemble GRCh38. Next, the alignment files were converted to a binary alignment map (BAM) files, sorted, and filtered to exclude reads with mapping quality scores <15. The retained reads were processed using SAMtools (v1.9) and Picard (v3.8; https://broadinstitute.github.io/picard/), which exclude low-quality reads and polymerase chain reaction duplicates, respectively. Finally, somatic SNV calling was performed for the whole-exome data from analysis-ready BAM files using Mutect2 (v3.8) for tumor samples, followed by filtering with a panel of 16 unmatched non-tumor leukocyte samples. Extensive filtering was applied to remove samples with a low mapping quality, as well as strand and position biases and oxoguanine oxidative artifacts. Residual germline mutations from the Genome Aggregation Database (https://gnomad.broadinstitute.org/) and Online Archive of Brazilian Mutations (http://abraom.ib.usp.br/) were removed. The raw sequencing data (fastq files) were deposited in the Sequence Read Archive (http://www.ncbi.nlm.nih.gov/sra; accession no. PRJNA505810).

### 4.3. Statistical Analyses

Data on baseline patient characteristics are expressed as absolute and relative frequencies for qualitative variables and as means ± standard deviations for quantitative variables. Mutational signature exposure and the TMB were considered to be continuous variables. Associations between qualitative variables were evaluated using the chi-squared test or Fisher’s exact test, as appropriate. Comparisons of the means of quantitative variables and groups were evaluated using the t-test or Mann–Whitney U test, as appropriate.

Overall survival functions were generated using the Kaplan–Meier estimator and compared between S^high^ and S^low^ groups using the log-rank test. A semiparametric Cox proportional-hazards model was fitted to the dataset to describe relationships between OS and the main clinical features. HRs and 95% CIs were calculated for all variables. A backward stepwise selection algorithm was applied, with different significance levels required to enter (*p* = 0.10) and remain in (*p* = 0.05) the model. Variables that acted as confounders (>20% change in coefficient) were also removed from the model. The proportional-hazards assumption was assessed based on the Schoenfeld residuals [57]. The analysis provided evidence that all the covariates had constant effects over time.

Multivariate analyses were performed to examine the main clinical features associated previously with OS (e.g., age, pathological stage, Lauren tumor subtype, and ethnicity), mutational signature exposures associated with dMMR, and molecular features (TMB and MSI status). Forest plots were created based on the results of the final multiple Cox regression model. Patients with metastasis were excluded from these analyses. In addition, we fitted simple and multiple logistic regression models to assess the effects of S2, S4, and S5 exposure on *MLH1* methylation. The overall performance, calibration, and discriminatory power of the final multiple logistic regression model were assessed using the Brier score, the Hosmer–Lemeshow goodness-of-fit test, and the AUC, respectively [58]. We also assessed the goodness of fit using a Q–Q plot. The significance level was fixed at 5% for all the tests (two-sided). Statistical analyses were performed using the R software (v3.5).

### 4.4. Mutational Signatures in Cell Lines

CRISPR-Cas9 knockout clones for *MLH1* were generated in human HAP1 cells using the guide RNA sequence 5′-AAGACAATGGCACCGGGATC-3′. Clonal populations with *MLH1* frameshift mutations were cultured for 3 months to allow for the accumulation of mutations during cellular division [59]. To identify mutations, genomic DNA was submitted to whole-genome sequencing. De novo somatic mutations, including substitutions, indels, and rearrangements, in subclones were identified by removing all mutations seen in parental clones. Next, SNVs were mapped onto trinucleotide sequences by including the 5′ and 3′ neighboring base contexts, followed by the estimation of the degree of sample exposure to previously detected mutational signatures [21].

### 4.5. Mutational Signature Estimation

All six classes of SNV (C > A, C > G, C > T, T > A, T > C, and T > G) from the public cohorts were mapped onto trinucleotide sequences by including the 5′ and 3′ neighboring base contexts. Next, the SNV spectrum with 96 trinucleotide mutation types for all samples from the public cohorts was loaded into signer [21] for the estimation of the optimal number of mutational signatures based on the median Bayesian information criterion. We next used cosine similarity (>0.7) to compare the de novo extracted mutational signatures with those described in the CS database (v2).

The SNV spectrum with 96 trinucleotide mutation types for samples from the validation cohort was loaded into signeR to estimate the degree of sample exposure to previously identified mutational signatures from the public cohorts. Samples exhibiting greater exposure (≥the third quartile level) to a given signature were assigned to the S^high^ groups and those with lesser exposure (<the third quartile level) were assigned to the S^low^ groups.

### 4.6. Molecular Features

We used the MSIseq [21] software for MSI status (MSI-H and non–MSI-H) prediction from whole-exome data. Briefly, this software is based on four machine-learning frameworks, and requires a catalog of sample somatic SNVs and microindels, a file containing the exact locations of mononucleotides (length ≥5 bases), and microsatellites consisting of di-, tri-, and tetranucleotide repeats, as annotated in the “simpleRepeats” track (http://hgdownload.cse.ucsc.edu/goldenpath/hg19/database). Consistent with the method proposed by Chalmers et al. [60], the TMB was calculated by dividing the total number of mutations by the length of the target region in megabases. Tumor heterogeneity was estimated using the “math.score” function in the “maftools” package (v3.8) [61]. Higher MATH scores indicate a greater heterogeneity. Available data on neoantigens for 77 TCGA-STAD samples were extracted (https://tcia.at/neoantigens) [62].

### 4.7. Identification of Significantly Mutated Genes

To assess the somatic mutation profiles of genes throughout the genome, we searched for genes that were mutated more frequently than expected by chance [27]. Pre-processed Mutation Annotation Format files (generated using the “prepareMutSig” function in “maftools” (v8) [27]) were loaded into the online MutSigCV server (v1.3.4; https://cloud.genepattern.org/gp/pages/index.jsf) [27]. Significantly mutated genes associated with S4 were evaluated to determine their associations with S4^high^, S4^low^, and subgroups defined by *MLH1* methylation status using the “compareMaf” function (“maftools” R package). The “somaticInteractions” function (“maftools” R package) was used to identify sets of genes that were mutually mutated in exclusive and co-occurring manner [29,61].

### 4.8. Evaluation of Tumor Microenvironment Composition and Immunological Aspects

We estimated cellular compositions from the TCGA bulk expression datasets using two complementary approaches. First, the CIBERSORT software, based on the deconvolution method for the characterization of cell composition in complex tissues from their gene expression profiles, was used [50]. CIBESORT takes advantage of a validated leukocyte gene signature matrix, termed LM22. This matrix contains 547 genes that distinguish 22 human hematopoietic cell phenotypes, including seven T cell types, naïve and memory B cells, plasma cells, NK cells, and myeloid subsets. Simultaneously, a recently introduced GSEA-based technique termed xCell [51] was used to infer 64 immune and stroma cell types. We used this approach to confirm the CIBERSORT findings and evaluate the stromal content.

A CIBERSORT analysis was performed online using a public server (http://cibersort.stanford.edu/) for the characterization of absolute and relative immune cell composition, with 1000 permutations and disabled quantile normalization as set parameters. Of the 380 TCGA-STAD tumor samples, 215 (56%) samples yielded data on infiltrating immune cells (CIBERSORT analysis, *p* < 0.05) and were included in further analysis (S4^high^, *n* = 50; S4^low^, *n* = 165). We also used xCell to confirm the results of the S4^high^ and S4^low^ sample comparison. xCell analysis was performed using the R package with default parameters (https://github.com/dviraran/xCell). To verify the immune effector responses in S4^high^ and S4^low^ samples, the differential expression of key immunoregulatory/inflammatory and cytotoxic markers was found. Group comparisons described in this section were performed using the Mann–Whitney *U* test with the significance level set to *p* < 0.05. We also used the pre-processed immune subtypes described by Thorsson et al. [34] for TCGA samples (available for 103 S4^high^ samples and 285 S4^low^ samples; Table S2).

## 5. Conclusions

In conclusion, whereas previous studies have aimed to classify patients using molecular and clinical features, such as the MSI status, TMB, and *MLH1* gene expression levels, this study provides evidence that classification based on mutational signature exposure can be used to identify groups of patients with common clinical, immunological, and mutational features related directly to better prognosis.

## Figures and Tables

**Table 1:** Clinicopathological features of gastric cancer samples according to mutational signature 4 DNA mismatch repair deficiency groups.

Variable	All (*n* = 787)		S4^low^ (*n* = 590)		S4^high^ (*n* = 197)		*p* Value
	N	%	N	%	N	%	
Age (mean ± SD)	64.17 ± 11.73		64.17 ± 11.73		66.25 ± 10.68		0.0024
Gender	767	97	571	91	196	99	
*Female*	274	0.36	192	0.34	82	0.42	0.0473
*Male*	493	0.64	379	0.66	114	0.58	
Race	726	92	546	93	180	91	
*White*	275	0.38	203	0.37	72	0.40	0.7427
*Black*	13	0.02	11	0.02	2	0.01	
*Asian*	437	0.60	331	0.61	106	0.59	
*Other*	1	0.00	1	0.00	0	0.00	
Anatomic Site	627	80	495	84	132	67	
*Cardia/Proximal*	168	0.27	148	0.30	20	0.15	0.0015
*Fundus/Body*	212	0.34	166	0.34	46	0.35	
*Antrum/Distal*	242	0.39	178	0.36	64	0.48	
*Other*	5	0.01	3	0.01	2	0.02	
Histology Lauren	467	59	383	65	84	43	
*Diffuse*	150	0.32	132	0.34	18	0.21	0.0041
*Intestinal*	301	0.64	235	0.61	66	0.79	
*Mixed*	16	0.03	16	0.04	0	0.00	
Stage T	712	90	526	89	186	94	
*T1-T2*	181	0.25	132	0.25	49	0.26	0.8116
*T3-T4*	531	0.75	394	0.75	137	0.74	
Stage N	712	90	526	59	186	94	
*N0*	173	0.24	115	0.22	58	0.31	0.0144
*N+*	539	0.76	411	0.78	128	0.69	
Stage M	707	90	524	89	183	93	
*M0*	623	0.88	461	0.88	162	0.89	0.9422
*M1*	62	0.01	47	0.09	15	0.08	
*MX*	22	0.03	16	0.03	6	0.03	
Pathological Stage	715	91	546	93	169	86	
*I*	85	0.12	58	0.11	27	0.16	0.0386
*II*	220	0.31	160	0.29	60	0.36	
*III*	289	0.40	228	0.42	61	0.36	
*IV*	121	0.15	100	0.18	21	0.12	
Molecular Subtype	403	51	289	49	114	58	
*CIN*	223	0.55	206	0.71	17	0.15	<0.0001
*GS*	50	0.12	47	0.16	3	0.03	
*EBV*	38	0.09	33	0.11	5	0.04	
*MSI*	85	0.21	0	0.00	85	0.75	
*POLE*	7	0.02	3	0.01	4	0.04	
MSIseq Status	787	100	590	100	197	100	
*MSI-H*	160	0.20	41	0.07	119	0.60	<0.0001
*Non MSI-H*	627	0.80	549	0.93	78	0.40	
Immune Subtypes	388	49	285	48	103	52	
*C1*	128	0.33	101	0.35	27	0.26	<0.0001
*C2*	209	0.54	135	0.47	74	0.72	
*C3*	35	0.09	34	0.12	1	0.01	
*C4*	9	0.02	8	0.03	1	0.01	
*C6*	7	0.02	7	0.02	0	0.00	

**Table 2:** Simple and multiple logistic regression results for *MLH1* methylation and DNA mismatch repair deficiency mutational signatures.

Variable	Coefficient	Standard Error	CI (95%) for Coefficient		*p*-Value	OR	CI (95%) for Coefficient	
			Lower	Upper			Lower	Upper
**Simple logistic regression model**								
ExpS2	4.772	0.5226	3.748	5.796	<0.0001	118.155	42.424	329.078
ExpS4	3.2424	0.3469	2.562	3.922	<0.0001	25.595	12.968	50.518
ExpS5	0.2725	0.1674	−0.056	0.601	0.104	1.313	0.946	1.823
**Multiple logistic regression model**								
Intercept	−2.640	0.300	−3.227	−2.052	<0.0001			
ExpS2	1.304	0.730	−0.126	2.733	0.074	3.682	0.881	15.386
ExpS4	3.1162	0.5348	2.068	4.164	<0.0001	22.561	7.909	64.353
ExpS5	−2.231	0.4067	−3.028	−1.434	<0.0001	0.107	0.048	0.238

## Data Availability

The raw sequencing data (fastq files) are openly and avaliable in the Sequence Read Archive (http://www.ncbi.nlm.nih.gov/sra; accession no. PRJNA505810).

## Supplementary Materials

The following are available online at https://www.mdpi.com/2072-6694/13/3/490/s1, Figure S1. Heatmap clustering of gastric cancer sample signature exposure. Mutational signatures called by signeR are arranged in rows, and samples are arranged in columns, Figure S2. Forest plot of hazard ratios for overall survival from univariate Cox model analysis. TMB, tumor mutational burden; MSI, microsatellite instability, Figure S3. Forest plot of hazard ratios for overall survival from the multivariate Cox model for (a) mutational signature S2 and (b) S5, Figure S4. Calibrated plots of 2-year survival (multiple Cox regression models) for (a) mutational signature S2, (b) S4, (c) S5, (d) high microsatellite instability, and (e) tumor mutational burden, Figure S5. Five-year overall survival for S4^high^ and S4^low^ groups in the public cohort, Figure S6. Five-year overall survival for S4^high^ and S4^low^ groups in the validation cohort, Figure S7. Scatter plots of Sperman’s correlation between MMR gene expression levels (FPKM) and methylation loads. Gene expression levels were plotted against average β values for the promoter-related CpG islands of MMR genes, Figure S8. Barplot showing the burdens of previously identified mutational signatures in the public gastric cancer cohort; isogenic wild-type cells are compared with two subclones with mutations in the *MLH1* gene induced by CRISP-Cas9 assay, Figure S9. Boxplots of immune and stroma cells scores, estimated by xCell (Aran et al., 2017), in the mutational signature S4^high^ and S4^low^ groups. * *p* < 0.05, ** *p* < 0.01, t test, Figure S10. Boxplots showing the normalized gene expression of immune-related genes. Immune gene markers were grouped as (a) inflammatory/cytotoxic, (b) suppressor/exhausted, (c) costimulatory/antigen presentation, and (d) other (natural killer cell receptors and monocyte/macrophage markers). Outlying values were removed. * *p* < 0.05, ** *p* < 0.01, *** *p* < 0.001, **** *p* < 0.0001, Mann–Whitney U test, Table S1. Significantly mutated genes in the mutational signature (S)4^high^ and S4^low^ groups and associated pathways. (a) Significantly mutated genes, related pathways, and frequency in the S4^high^ group. (b) Description of pathways found for the S4^high^ gene set. (c) Significantly mutated genes, related pathways and frequency in the S4^low^ group. (d) Description of pathways found for the S4^low^ gene set, Table S1 SignificantlyMutatedGenes-Pathways.xlsx, Table S2. Metadata on sample clinical, molecular, and immune features, Table S2 Clinical-Molecular-features.xlsx, Table S3. Clinicopathological features of gastric cancer samples by cohort, Table S4. Clinicopathological features of gastric cancer samples according to mutational signature 4 DNA mismatch repair deficiency in the validation cohort.

## Abbreviations

CIN	chromosomal instability
dMMR	DNA mismatch repair deficiency
HRR	homologous recombination repair
GS	genomically stable
GSEA	gene set enrichment analysis
MSI	microsatellite instability
MSI-H	microsatellite instability high
MSI-L	microsatellite instability low
NER	nucleotide excision repair
OS	overall survival
SNV	single-nucleotide variation
TCGA	The Cancer Genome Atlas
TMB	tumor mutational burden
