# Supplementary material for: Mutational Signatures Driven by Epigenetic Determinants Enable the Stratification of Patients with Gastric Cancer for Therapeutic Intervention
Source: Cancers (Basel). 2021 Jan 27;13(3):490. doi: 10.3390/cancers13030490 (PMC7866019; doi:10.3390/cancers13030490)
Supplement: Supplementary file 1 [file cancers-13-00490-s001.zip › cancers-1041497-supplementary material-.pdf]

Figure S1: **Heatmap clustering of gastric cancer sample signature exposure**. Mutational signatures called by signeR are arranged in rows, and samples are arranged in columns.

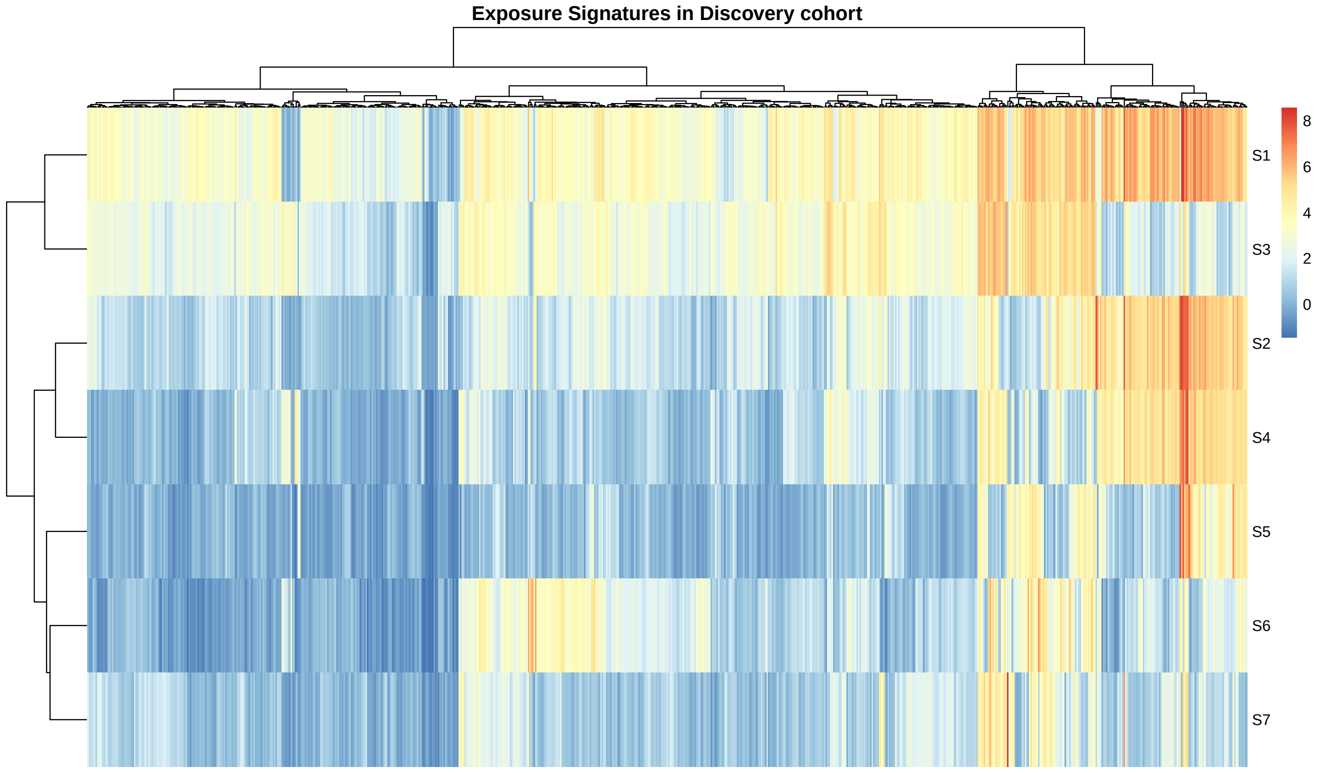

Figure S2: **Forest plot of the hazard ratios for overall survival from univariate Cox model analysis.** TMB, tumor mutational burden; MSI, microsatellite instability.

Variable	N (total)	N (deaths)	Hazard Ratio [95% CI]	P−Value
Age	581	224	1.1906 [1.038,1.366]	0.013 *
Race (White)	539	206	1.4551 [1.104,1.918]	0.008 **
Lauren's Histology (Intestinal)	317	124	0.8441 [0.577,1.234]	0.382
Tumor Site (Cardia/Proximal)	584	224	1.2932 [0.943,1.773]	0.11
Stage				
II	529	206	1.6673 [0.956,2.909]	0.072
III	529	206	2.5245 [1.475,4.322]	0.001 **
TMB	584	224	0.675 [0.495,0.92]	0.013 *
MSI−high	584	224	0.7389 [0.513,1.065]	0.105
Molecular Subtype				
EBV	357	134	1.0694 [0.595,1.923]	0.823
GS	357	134	0.8094 [0.466,1.405]	0.452
MSI	357	134	0.6085 [0.378,0.979]	0.04 *
POLE	357	134	1.675 [0.409,6.859]	0.473
Mutational signatures exposure				
S1	584	224	0.7235 [0.557,0.94]	0.015 *
S2	584	224	0.7849 [0.61,1.01]	0.059
S3	584	224	0.8678 [0.739,1.019]	0.084
S4	584	224	0.6393 [0.428,0.956]	0.029 *
S5	584	224	0.3387 [0.124,0.923]	0.034 *
S6	584	224	0.8642 [0.674,1.108]	0.25
S7	584	224	0.8873 [0.708,1.112]	0.299

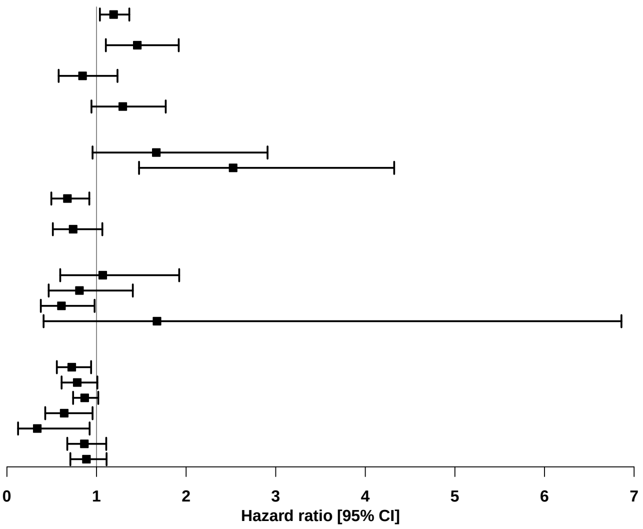

Figure S3: **Forest plot of hazard ratios for overall survival from the multivariate Cox model for (a) mutational signature (S) 2 and (b) S5.**

(a) **Signature S2**

Variable	N (total)	N (deaths)	Hazard Ratio [95% CI]	P−Value
Age	484	188	1.2427 [1.059,1.459]	0.008 **
Race (White)	484	188	1.449 [1.082,1.941]	0.013 *
Stage				
II	484	188	1.8326 [0.987,3.401]	0.055
III	484	188	2.8782 [1.575,5.261]	0.001 **
S2	484	188	0.7758 [0.595,1.011]	0.06

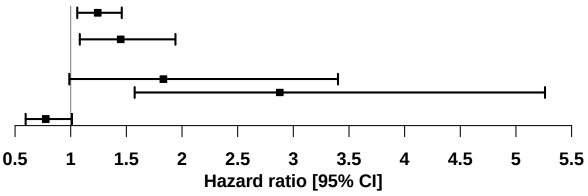

(b) **Signature S5**

Variable	N (total)	N (deaths)	Hazard Ratio [95% CI]	P−Value
Age	484	188	1.2297 [1.049,1.442]	0.011 *
Race (White)	484	188	1.3738 [1.024,1.843]	0.034 *
Stage				
II	484	188	1.8271 [0.984,3.392]	0.056
III	484	188	2.8944 [1.583,5.291]	0.001 **
S5	484	188	0.358 [0.114,1.127]	0.079

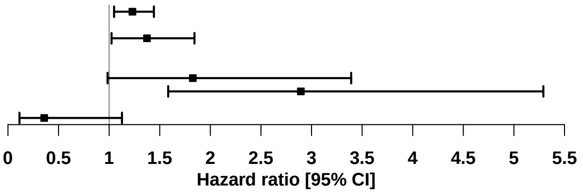

Figure S4: **Calibrated plots of 2-year survival (multiple Cox regression models) for (a) mutational signature (S)2, (b) S4, (c) S5, (d) high microsatellite instability, and (e) tumor mutational burden.**
.

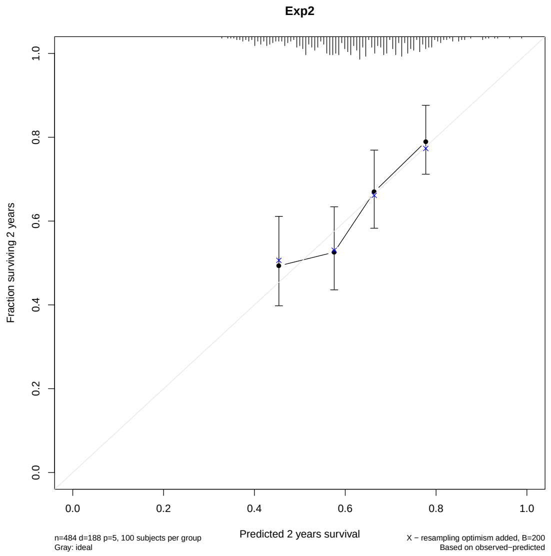

(a) **Mutational signature S2**

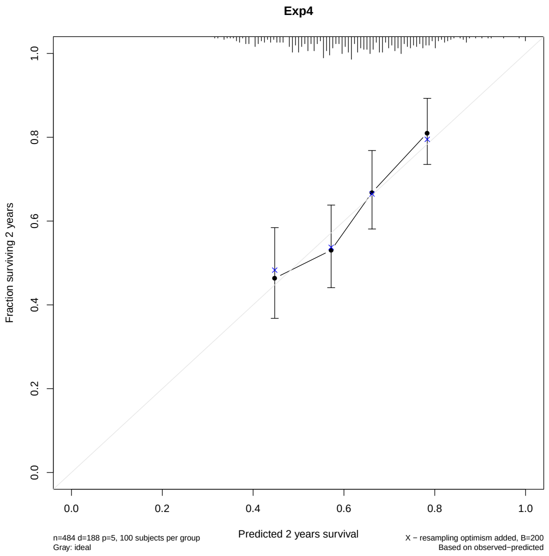

(b) **Mutational signature S4**

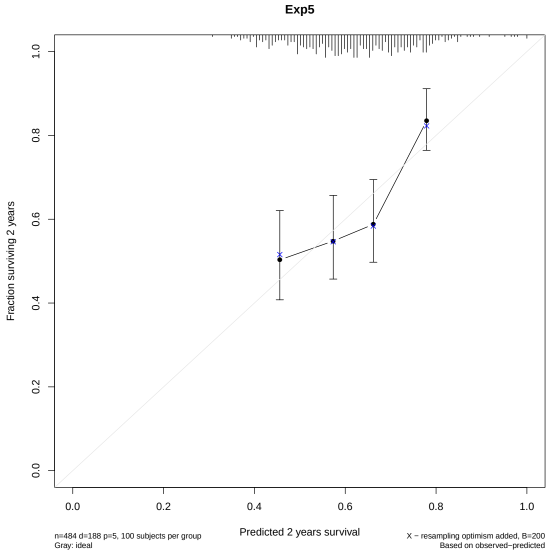

(c) **Mutational signature S5**

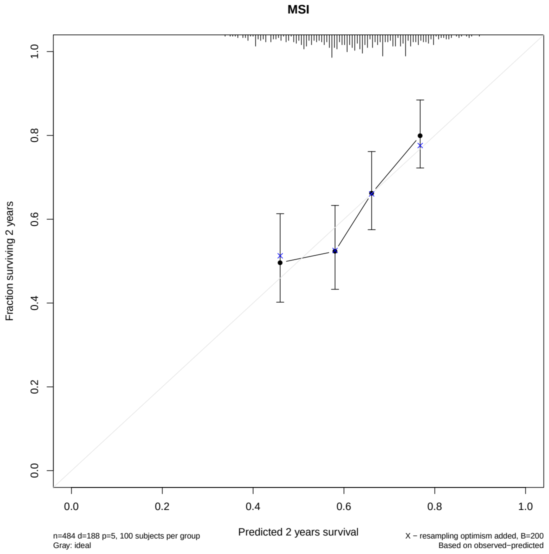

(d) **MSI-high status**

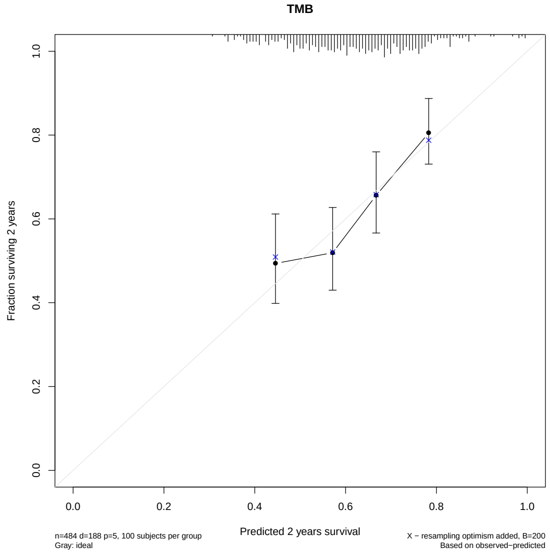

(e) **Tumor Mutational Burden**

Figure S5: **Five-year overall survival for S4^{high} and S4^{low} groups in the public cohort.**

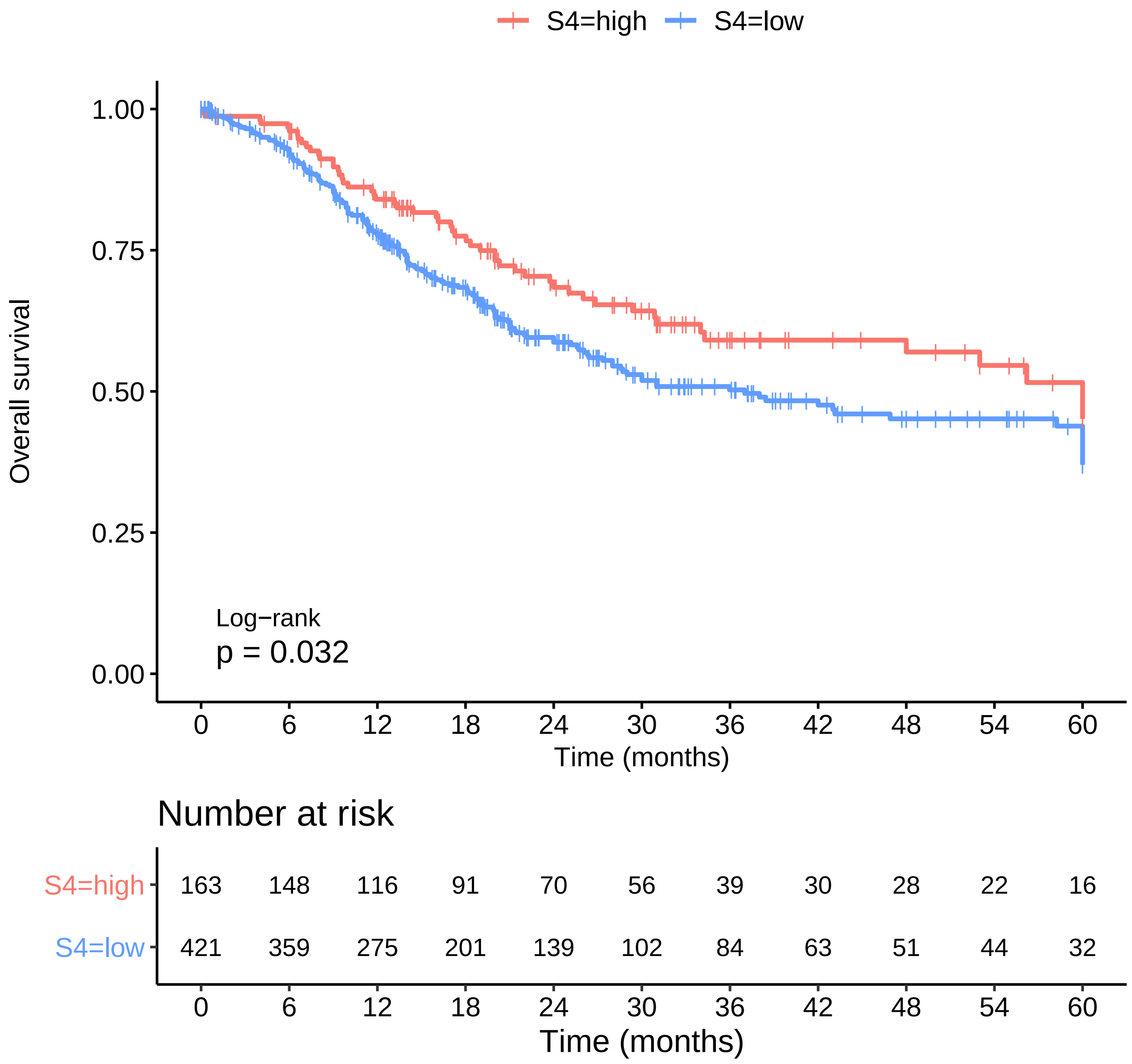

Figure S6: **Five-year overall survival for S4^{high} and S4^{low} groups in the validation cohort.**

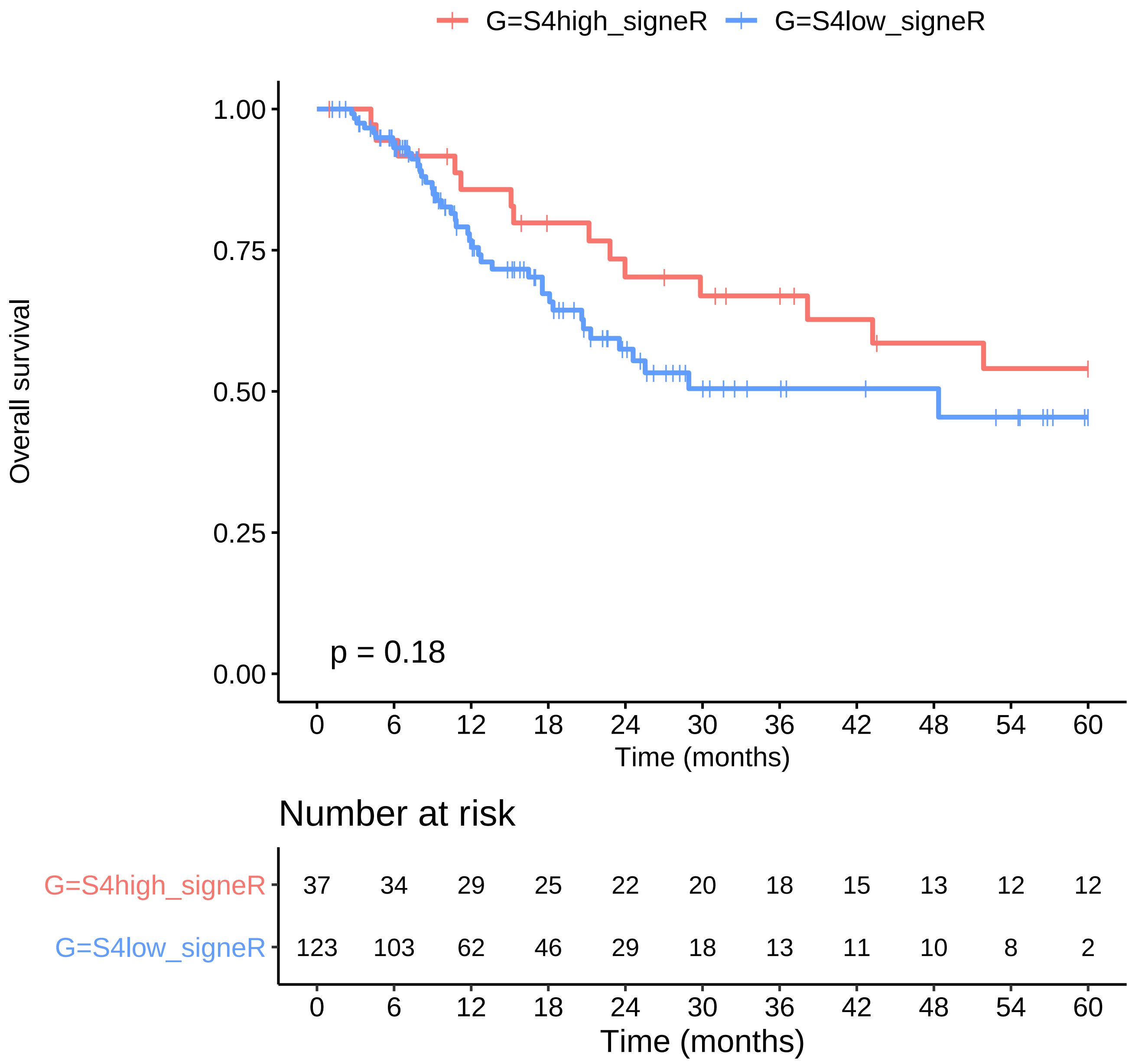

Figure S7: **Scatter plots of Spearman's correlation between MMR gene expression levels - FPKM (log-scaled) and methylation loads**. Gene expression levels were plotted against average β values for the promoter-related CpG islands of MMR genes.

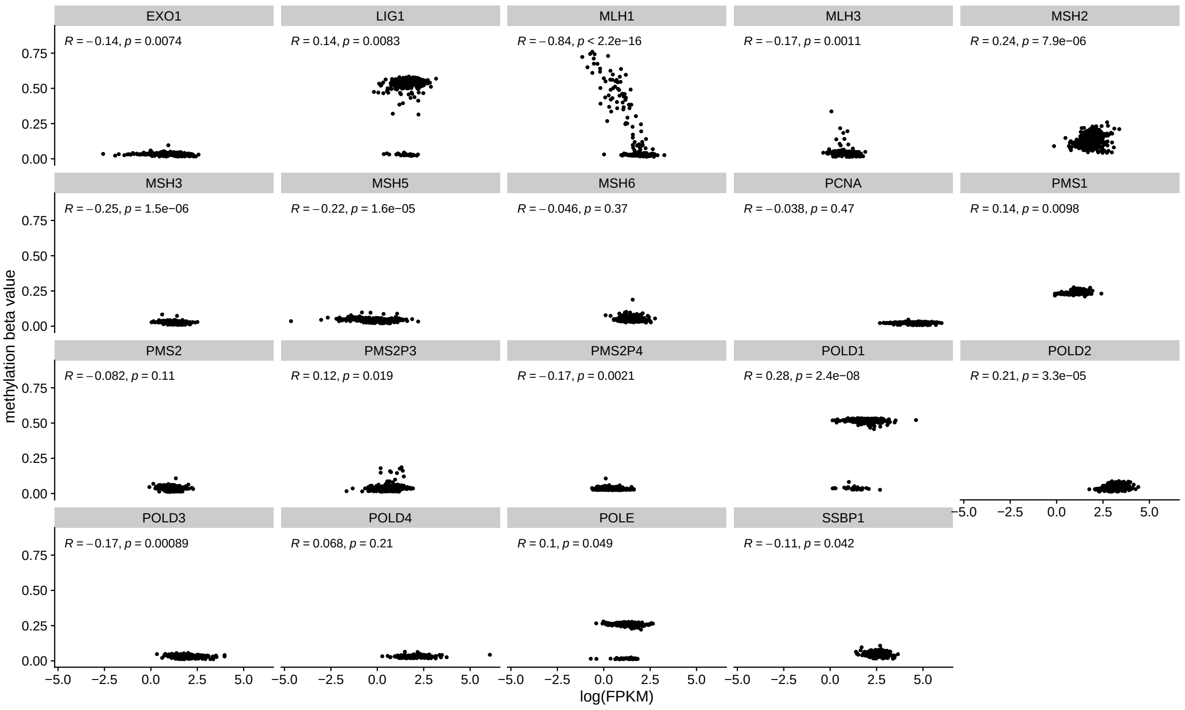

Figure S8: **Barplot showing the burdens of previously identified mutational signatures previously identified in the public gastric cancer cohort; isogenic wild-type cells are compared with two subclones with mutations in the *MLH1* gene induced by CRISP-Cas9 assay.**

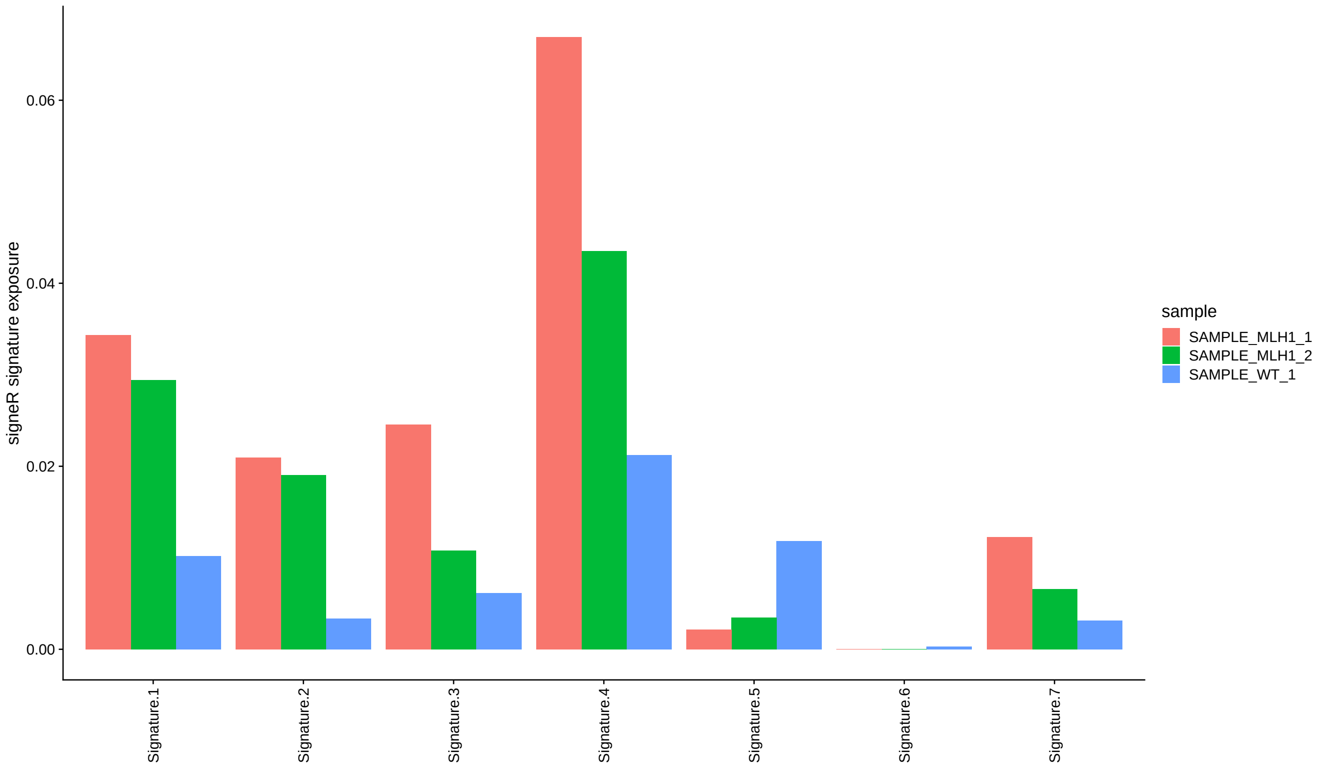

Figure S9: **Boxplots of immune and stroma cells scores, estimated by xCell [?], in the mutational signature (S)4^{high} and S4^{low} groups.** $*p < 0.05$, $**p < 0.01$, *t test.*

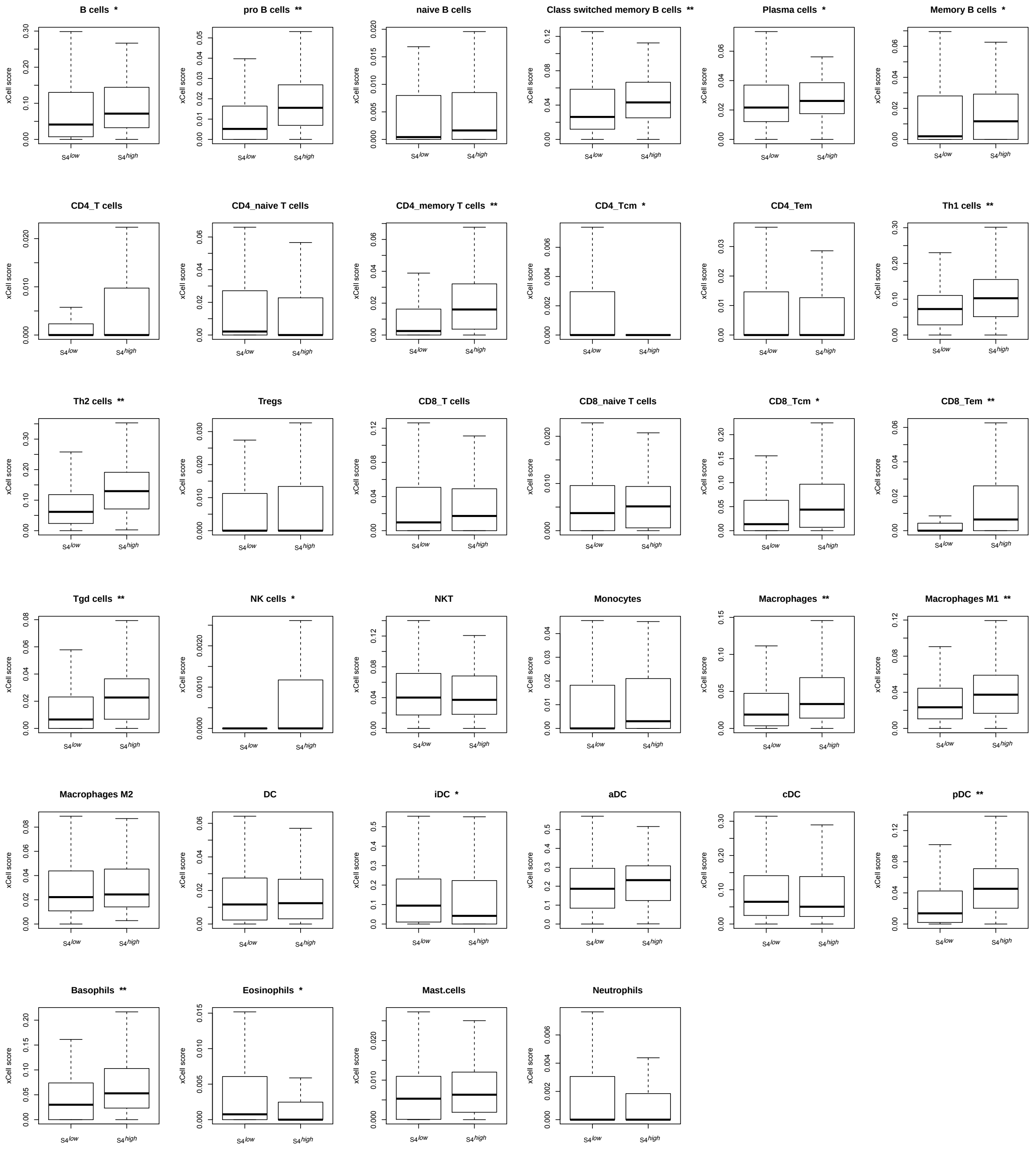

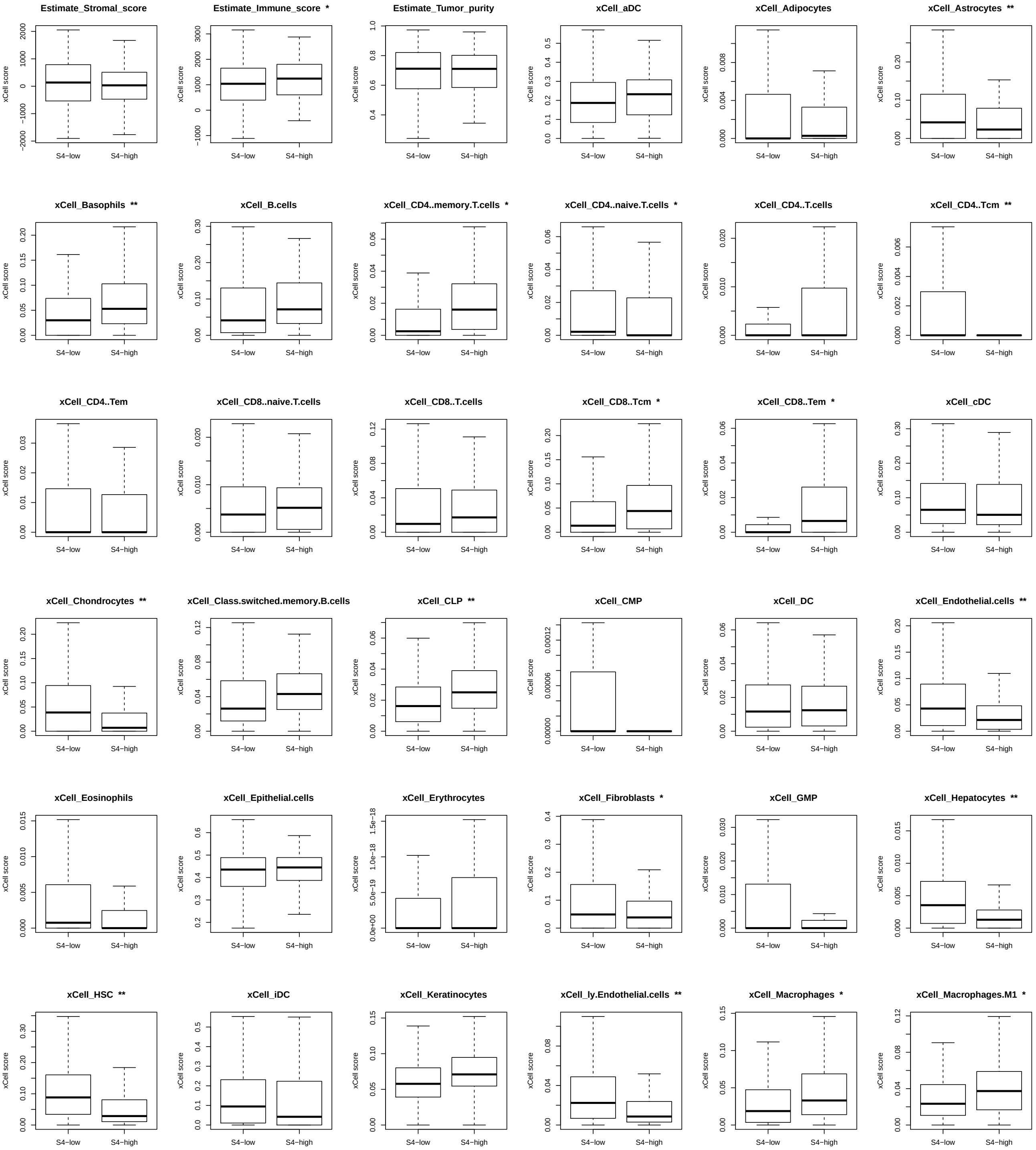
Estimate_Stromal_score
Estimate_Immune_score *
Estimate_Tumor_purity
xCell_aDC
xCell_Adipocytes
xCell_Astrocytes **
xCell_Basophils **
xCell_B.cells
xCell_CD4..memory.T.cells *
xCell_CD4..naive.T.cells *
xCell_CD4..T.cells
xCell_CD4..Tcm **
xCell_CD4..Tem
xCell_CD8..naive.T.cells
xCell_CD8..T.cells
xCell_CD8..Tcm *
xCell_CD8..Tem *
xCell_cDC
xCell_Chondrocytes **
xCell_Class.switched.memory.B.cells
xCell_CLP **
xCell_CMP
xCell_DC
xCell_Endothelial.cells **
xCell_Eosinophils
xCell_Epithelial.cells
xCell_Erythrocytes
xCell_Fibroblasts *
xCell_GMP
xCell_Hepatocytes **
xCell_HSC **
xCell_iDC
xCell_Keratinocytes
xCell_ly.Endothelial.cells **
xCell_Macrophages *
xCell_Macrophages.M1 *
xCell score
S4−low
S4−high

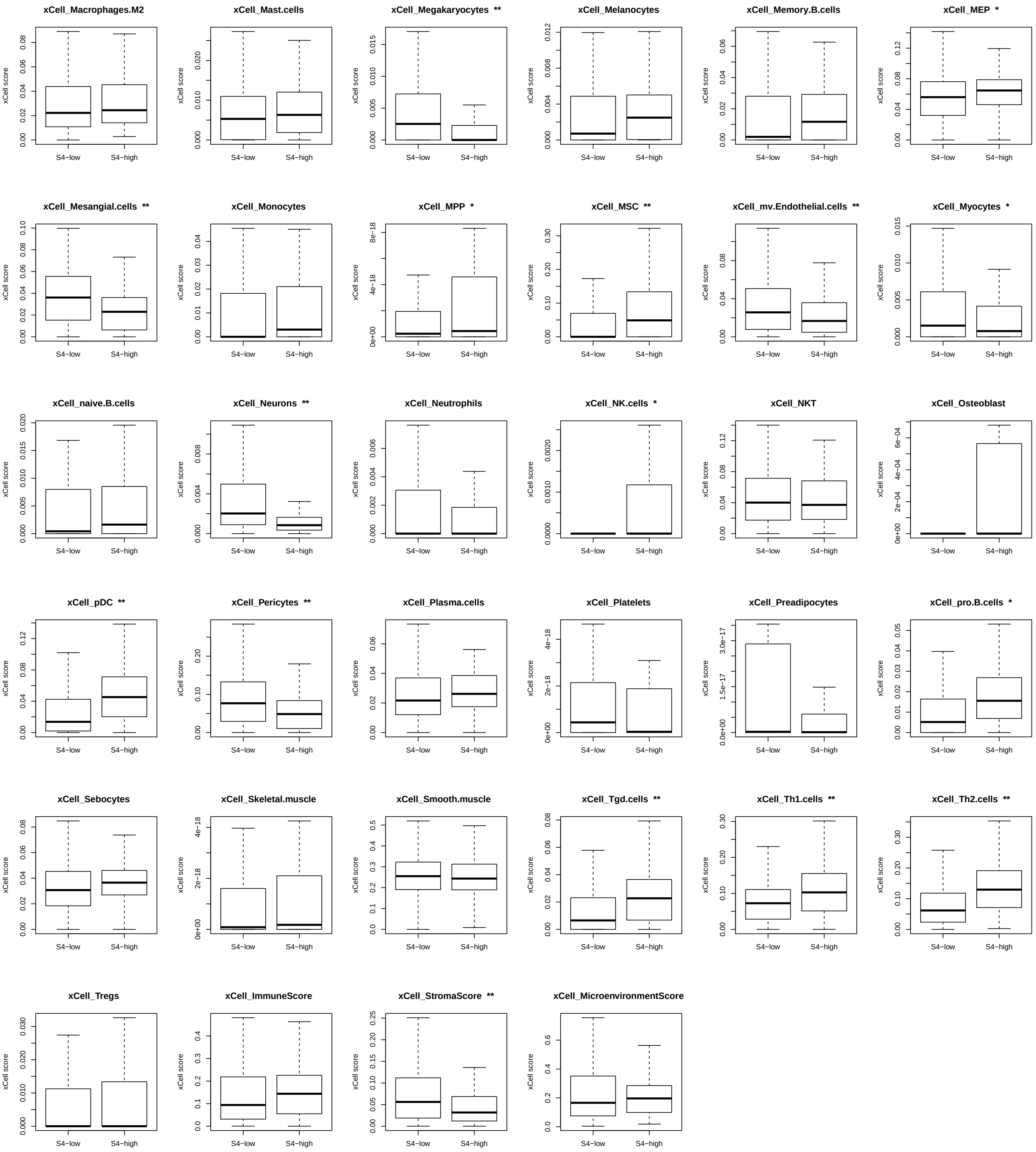
xCell_Macrophages.M2
xCell_Mast.cells
xCell_Megakaryocytes **
xCell_Melanocytes
xCell_Memory.B.cells
xCell_MEP *
xCell_Mesangial.cells **
xCell_Monocytes
xCell_MPP *
xCell_MSC **
xCell_mv.Endothelial.cells **
xCell_Myocytes *
xCell_naive.B.cells
xCell_Neurons **
xCell_Neutrophils
xCell_NK.cells *
xCell_NKT
xCell_Osteoblast
xCell_pDC **
xCell_Pericytes **
xCell_Plasma.cells
xCell_Platelets
xCell_Preadipocytes
xCell_pro.B.cells *
xCell_Sebocytes
xCell_Skeletal.muscle
xCell_Smooth.muscle
xCell_Tgd.cells **
xCell_Th1.cells **
xCell_Th2.cells **
xCell_Tregs
xCell_ImmuneScore
xCell_StromaScore **
xCell_MicroenvironmentScore
xCell score
S4−low
S4−high

Figure S10: **Boxplots showing the normalized gene expression of immune-related genes.** Immune gene markers as (a) inflammatory/cytotoxic, (b) suppressor/exhausted, (c) costimulatory/antigen presentation, and (d) other natural killer cell receptors and monocyte/macrophage markers. Outliying values were removed.$*p < 0.05$, $**p < 0.01$, $***p < 0.001$, $****p < 0.0001$, Mann-Whitney U test.

(a)

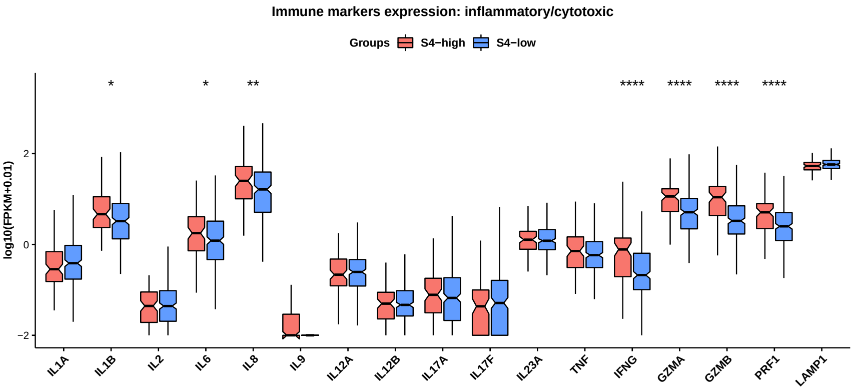

(b)

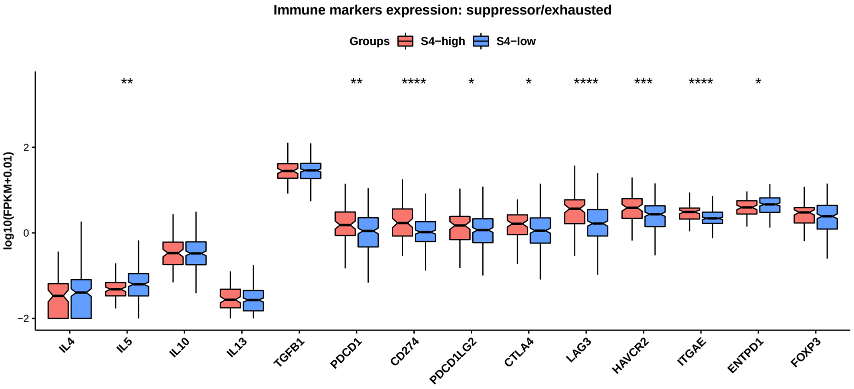

(c)

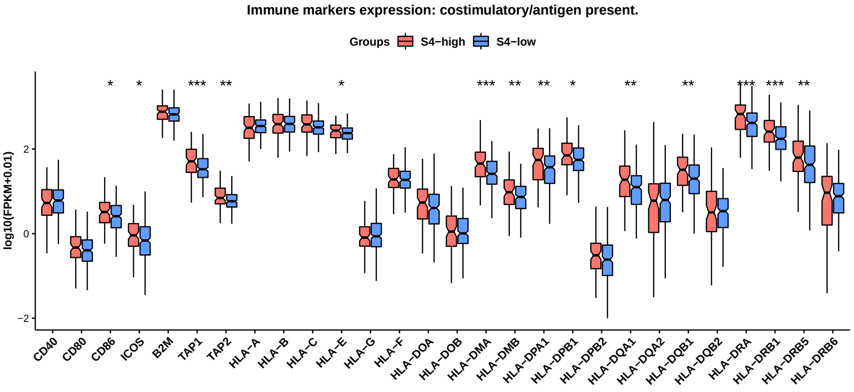

(d)

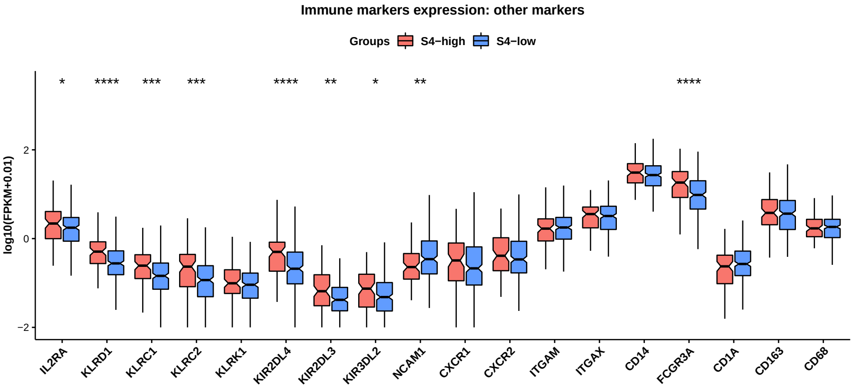

Table S1: **Metadata on sample clinical, molecular and immune features.**

TableS1_Clinical-Molecular-features.xlsx.

Table S2: **Significantly mutated genes in the mutational signature (S)4^{high} and S4^{low} groups and associated pathways.** (a) Significantly mutated genes, related pathways and frequency in the S4^{high} group. (b) Description of pathways found for the S4^{high} gene set. (c) Significantly mutated genes, related pathways and frequency in the S4^{low} group. (d) Description of pathways found for the S4^{low} gene set.

TableS2_SignificantlyMutatedGenes-Pathways.xlsx.

Table S3: **Clinicopathological features of gastric cancer samples by cohort.**

	$TCGA(n = 439)$		$cBioPortal(n = 226)$		$ICGC(n = 122)$	
	N	%	N	%	N	%
**Age (mean ± SD)**	65.75 ± 10.69		63.1 ± 13.73		60.30 ± 10.74	
**Gender**	439	100	206	91	122	100
Female	158	36	79	38	37	30
Male	281	64	127	62	85	70
**Race**	378	86	226	100	122	100
White	275	73	0	0	0	0
Black	13	3	0	0	0	0
Asian	89	24	226	100	122	100
Other	1	0.26	0	0	0	0
**Anatomic Site**	422	96	205	91	0	0
Cardia/Proximal	107	25	61	30	0	0
Fundus/Body	151	36	61	30	0	0
Antrum/Distal	159	38	83	40	0	0
Other	5	1	0	0	0	0
**Histology Lauren**	261	59	206	91	0	0
Diffuse	73	28	77	37	0	0
Intestinal	188	72	113	55	0	0
Mixed	0	0	16	8	0	0
**Stage T**	430	98	156	69	117	96
$T1 - T2$	115	27	42	27	24	20
$T3 - T4$	315	73	114	73	93	80
**Stage N**	421	96	156	69	117	96
$N0$	131	31	20	13	22	19
$N+$	290	69	136	87	95	81
**Stage M**	439	100	156	69	112	92
$M0$	387	88	133	85	103	92
$M1$	30	7	23	15	9	8
MX	22	5	0	0	0	0
**Pathological Stage**	413	94	205	90	97	79
I	61	15	15	7	97	79
II	165	40	38	18	17	17
III	157	38	72	35	60	62
IV	30	7	80	39	11	11
**Molecular Subtype**	383	87	20	9	0	0
CIN	223	58	0	0	0	0
GS	50	13	0	0	0	0
EBV	30	8	8	40	0	0
MSI	73	9	12	60	0	0
$POLE$	7	2	0	0	0	0
**MSIseq Status**	439	100	226	100	122	100
$MSI - high$	87	20	45	20	28	23
$nonMSI - high$	352	80	181	80	94	77
**Immune Subtype**	388	88	0	0	0	0
$C1$	128	33	0	0	0	0
$C2$	209	54	0	0	0	0
$C3$	35	9	0	0	0	0
$C4$	9	2	0	0	0	0
$C6$	7	2	0	0	0	0

Table S4: **Clinicopathological features of gastric cancer samples according to mutational signature S4 DNA mismatch repair deficiency in the validation cohort**

	$All (n = 170)$		$S4^{low} (n = 127)$		$S4^{high} (n = 43)$		$Pvalue$
	N	%	N	%	N	%	
**Age (mean ± SD)**	61.06 ± 13.33		60.182 ± 13.24		63.65 ± 13.39		**0.0726**
**Gender**	170	100	127	100	43	100	
Female	64	38	45	35	19	44	0.3999
Male	106	62	82	65	24	56	
**Race**	154	91	120	94	34	79	
White	91	59	72	60	19	56	0.7449
Black	11	7	7	6	4	12	
Asian	20	13	16	13	4	12	
Brown	31	20	24	20	7	21	
Other	1	1	1	1	0	0	
**Anatomic Site**	170	100	127	100	43	100	
Cardia/GEJ/Distalesophagus	57	34	47	37	10	23	**0.0396**
Anastomosicmouth	3	2	3	2	0	0	
Body	62	36	48	38	14	33	
Antrum	44	26	28	22	16	37	
Linitis	4	2	1	1	3	7	
**Histology Lauren**	152	89	111	87	41	95	
Diffuse	74	49	58	52	16	39	0.2246
Intestinal	59	39	39	35	20	49	
Mixed	19	13	14	13	5	12	
**Stage T**	103	89	67	53	36	84	
$T1 - T2$	44	43	31	46	13	36	0.5326
$T3 - T4$	59	57	36	54	23	64	
**Stage N**	102	60	75	59	37	86	
$N0$	43	42	39	52	14	38	0.226
$N+$	59	57	36	48	23	62	
**Stage M**	111	65	74	58	37	86	
$M0$	27	24	4	5	23	62	<**0.0001**
$M1$	2	2	2	3	0	0	
MX	82	74	68	92	14	38	
**Pathological Stage**	105	62	68	53	37	86	
I	31	30	24	35	7	19	0.2194
II	35	33	23	34	12	32	
III	31	30	17	25	14	38	
IV	8	8	4	6	4	11	
**MSIseq Status**	170	100	127	100	43	100	
$MSI - high$	38	22	2	2	36	84	<**0.0001**
$nonMSI - high$	132	78	125	98	7	16	
